# Radium-223 decreases PD-L1 expression in bone metastases: A study with [^89^Zr]Zr-atezolizumab PET and immunohistological correlation

**DOI:** 10.1007/s00259-026-08175-9

**Published:** 2026-09-12

**Authors:** Sui Wai Ling, Anouk de Jong, Mark Gorris, Marcel Segbers, Laura Graven, Marjolein van Driel, Geert van Leenders, Khrystany Isebia, Marjolijn Lub – de Hooge, Paul Hamberg, Niven Mehra, Mandy van Brakel, Ronald de Wit, Reno Debets, Elisabeth de Vries, Tessa Brabander, Jolanda de Vries, Astrid van der Veldt

**Affiliations:** 1 Department of Radiology and Nuclear Medicine, Erasmus MC, Rotterdam, the Netherlands; 2 Department of Medical Oncology, Erasmus MC Cancer Institute, Doctor Molewaterplein 40 3015 GD, Rotterdam, the Netherlands; 3 Department of Medical BioSciences, Radboudumc, Nijmegen, the Netherlands; 4 Department of Internal Medicine, Erasmus MC, Rotterdam, the Netherlands; 5 Department of Pathology, Erasmus MC Cancer Institute, Rotterdam, the Netherlands; 6 Department of Clinical Pharmacy and Pharmacology, UMC Groningen, University of Groningen, Groningen, the Netherlands; 7 Department of Medical Oncology, UMC Groningen, University of Groningen, Groningen, the Netherlands; 8 Department of Internal Medicine, Franciscus Gasthuis and Vlietland Hospital, Rotterdam, the Netherlands; 9 Department of Medical Oncology, Radboud UMC, Nijmegen, the Netherlands

**Keywords:** Radium-223, Gallium-68, PSMA, Zirconium-89, Atezolizumab

## Abstract

**Purpose:**

Most patients with metastatic castration-resistant prostate cancer (mCRPC) do not benefit from treatment with immune checkpoint inhibitors targeting programmed cell death (ligand) 1 (PD-(L)1). Radiation might improve response to immunotherapy by increasing the immunogenicity of prostate cancer. The objective of this study was to investigate radium-223-mediated changes in the micro- and macroenvironment of mCRPC.

**Methods:**

In this prospective, multicenter study (ICTRP NL-OMON26890), 28 patients with mCRPC and predominantly bone metastases were treated with radium-223, according to standard-of-care. Primary endpoint was failure-free survival (FFS). Imaging included sequential positron emission tomography (PET) with gallium-68 prostate-specific membrane antigen (^68^Ga-PSMA) and zirconium-89 atezolizumab (^89^Zr-atezolizumab) to quantify whole-body expression of PD-L1 in vivo (SUV_mean/max_). Paired PET analysis included nine patients. The immune status of patients was characterized in biopsies from bone metastases and in blood using (multiplex) immunohistochemistry (IHC) and flow cytometry, respectively. Sequential biopsy analysis included two patients.

**Results:**

^89^Zr-atezolizumab uptake in ^68^Ga-PSMA positive bone metastases showed high intra- and inter-patient heterogeneity. In the total volume of ^68^Ga-PSMA-positive bone lesions, the median SUV_mean_ of ^89^Zr-atezolizumab was 2.5 (IQR 0.9–3.4) at baseline and 0.9 (IQR 0.5–2.5) after three cycles of radium-223, which was higher than the physiological uptake in bone marrow at baseline and after treatment with radium-223. After three cycles of radium-223, ^89^Zr-atezolizumab uptake decreased in bone metastases, independent of response on ^68^Ga-PSMA PET, and the number of lymphocytes in tumor tissue and blood decreased concomitantly.

**Conclusions:**

Treatment of mCRPC bone metastases with radium-223 results in a significant decrease of in vivo PD-L1-expression on PET imaging and a decrease in immune cells in bone metastases and blood. These findings indicate that treatment with alpha emitting radium-223 alters the immune microenvironment, thereby potentially reducing the efficacy of PD-(L)1 inhibitors.

**Supplementary Information:**

The online version contains supplementary material available at https://doi.org/10.1007/s00259-026-08175-9.

## Translational relevance

Most patients with metastatic castration-resistant prostate cancer (mCRPC) do not benefit from treatment with immune checkpoint inhibitors targeting programmed cell death (ligand) 1 (PD-(L)1). By increasing the immunogenicity of the immunosuppressive prostate cancer microenvironment, radiation might improve response to immunotherapy. Therefore, we aimed to gain insight into the immune response to the radionuclide radium-223.

In this prospective study, 28 patients with mCRPC were treated with radium-223. Using positron emission tomography (PET) with gallium-68 prostate-specific membrane antigen (68Ga-PSMA) and zirconium-89 atezolizumab (^89^Zr-atezolizumab), we showed that whole-body expression of PD-L1 decreased after treatment with radium-223. In addition, the number of lymphocytes in tumor tissue and blood decreased. These findings indicate that treatment with alpha emitting radium-223 negatively affects the immune microenvironment, thereby impeding treatment with PD-(L)1 inhibitors.

**Supplementary Information:**

The online version contains supplementary material available at https://doi.org/10.1007/s00259-026-08175-9.

## Background

With 6.8% of all cancer-related deaths, prostate cancer is the fifth most common cause of death due to cancer among men worldwide [1]. Despite treatment with taxane-based chemotherapy, second-generation androgen receptor signaling inhibitors, PARP-inhibitors, and radionuclide therapies, the median 5-year survival of patients with metastatic prostate cancer is 32% [2].

In the last decade, the introduction of immune checkpoint inhibitors targeting PD-1/PD-L1 has improved the overall survival (OS) of patients with several tumor types [3–8]. However, durable responses to immune checkpoint inhibition were only reported in small subsets of patients with metastatic castration resistant prostate cancer (mCRPC) [9–11]. This poor response to immune checkpoint inhibitors is due to the low tumor mutational burden and suppressive immune microenvironment of prostate cancer [12, 13].

To overcome the suppressive immune microenvironment of prostate cancer, immunotherapy has been combined with other treatments, including radiotherapy [14]. Radiation may increase the immunogenicity of the tumor through direct cell damage and induction of inflammation, characterized by the secretion of inflammatory cytokines, the recruitment of dendritic cells, and activation of anti-tumor T cells. In addition, localized radiation at one tumor site may result in an anti-tumor response at other tumor sites, a phenomenon known as the abscopal effect [15]. In clinical trials, enhanced clinical antitumor activity was observed when immune checkpoint inhibitors were combined with external radiation [16–18].

In contrast to external radiotherapy, intravenously administered radionuclides can target multiple metastatic sites. The alpha emitting radionuclide radium-223 is approved for patients with mCRPC who have predominantly bone metastases without visceral metastases. In the phase III ALSYMPCA trial, radium-223 improved OS by 3.6 months in patients with mCRPC, compared to patients who received placebo with best standard of care [19].

Comparable to external radiation, treatment with radium-223 might sensitize prostate cancer for immunotherapy [20]. In vitro, radium-223 enhances T cell-mediated lysis of prostate cancer cells by inducing expression of MHC-I and calreticulin, which are important proteins for efficient antigen processing and presentation [21]. In patients, treatment with radium-223 resulted in enriched pathways of DNA damage repair in plasma exosomes with high exosomal expression levels of programmed cell death ligand 1 (PD-L1), lymphocyte-activation gene 3 (LAG-3) and indoleamine 2,3-dioxygenase (IDO) resulting in suppression of the immune system, which correlated with poorer survival [22]. Subsequent exploration in a prostate cancer mouse model also revealed induction of PD-L1 in the tumor after treatment with radium-223. In addition, mice, treated with radium-223 and immune checkpoint inhibition (anti-PD-1 and anti-CTLA-4) showed higher tumor regression than those treated with either anti-PD-1 or anti-CTLA-4 [22].

In tumor tissue, PD-L1-expression can be measured using immunohistochemistry (IHC), but predicting response to immune checkpoint inhibitors (anti-PD-1) is still controversial [23, 24]. In contrast to a single biopsy, positron emission tomography / computed tomography (PET/CT) using the PD-L1 antibody ^89^Zr-atezolizumab can quantify PD-L1-expression in the whole body in vivo [25].

To assess a rationale for the combination of radium-223 and immune checkpoint inhibition, we investigated the immune response to radium-223 in patients with mCRPC. To this end, we investigated PD-L1-expression by sequential [^89^Zr]Zr-atezolizumab PET and IHC of tumor tissue, together with the quantification of immune cell populations in blood and in tumor tissue before, during and after treatment with radium-223.

## Methods

### Study design

In this prospective, multicenter biomarker discovery study (International Clinical Trials Registry Platform NL-OMON26890/Netherlands Trial Register NL7380, Radium223Insight), patients were included in the Erasmus Medical Center (EMC) Cancer Institute, Franciscus Gasthuis & Vlietland (FGV), and Radboud University Medical Center (RUMC), the Netherlands. The study was conducted in accordance with the declaration of Helsinki and approved by the institutional review boards of all three hospitals (MEC 18-1562). All patients gave written informed consent before any study procedure. Patients were scheduled to receive six consecutive injections of radium-223 with an interval of four weeks, according to standard-of-care. Study procedures included sequential blood draws, biopsies of bone metastases, and the acquisition of images by different modalities (Fig. S1). The study was described according to the STROBE statement.

## Eligibility

Patients with mCRPC and predominantly bone metastases were eligible if they had progressive disease, had received at least two prior treatment lines (unless the patient was not able and/or unwilling to receive other treatments), and had not received prior chemotherapy other than docetaxel. Full in- and exclusion criteria are described in Supplemental Methods 1.1.

## Study endpoints

The primary endpoint was failure-free survival (FFS), defined as time from start of radium-223 to the next line of treatment, best supportive care, or death. Next line of treatment or best supportive care started upon clinical, biochemical, and/or radiological signs of progressive disease according to the Prostate Cancer Working Group 3 (PCWG3) criteria [26]. Good and poor responders were defined as patients with FFS of ≥ 24 weeks or < 24 weeks, i.e., time for completing six cycles of radium-223, respectively. To avoid bias in treatment decisions, treating physicians were blinded to the results of all study-related procedures, including imaging with [^68^Ga]Ga-PSMA PET/CT and [^89^Zr]Zr-atezolizumab PET/CT. Only the baseline [^68^Ga]Ga-PSMA PET/CT was not blinded since this scan was required to exclude visceral disease according to the inclusion criteria. Detection of visceral metastases or other significant findings (e.g. bone fractures) was reported by the nuclear medicine physician to the treating physician.

Here, we report the primary endpoint and parameters based on sequential measurements including [^68^Ga]Ga-PSMA PET/CT, [^89^Zr]Zr-atezolizumab PET/CT, (multiplex) IHC of tumor tissue, and immune cells and their subsets in blood. At different time points, immune response-related parameters were evaluated for changes over time in the complete patient cohort and in subgroups of patients with a good and poor response. Results of response evaluation with [^68^Ga]Ga-PSMA PET/CT and liquid biopsies were previously reported^28,29^.

## Study procedures

All imaging and biopsy procedures were performed at EMC and RUMC. Patients from the FGV were referred to the EMC for these study procedures.

## PET imaging with [^68^Ga]Ga-PSMA and [^89^Zr]Zr-atezolizumab

[^68^Ga]Ga-PSMA PET/CT was performed at baseline, after three and six cycles of radium-223 and at time of treatment failure, while [^89^Zr]Zr-atezolizumab PET/CT was performed at baseline and after three cycles of radium-223 (Supplemental Methods 1.2). Production, administration and image acquisition of [^68^Ga]Ga-PSMA-11 were previously described [27]. [^89^Zr]Zr-atezolizumab was produced at the University Medical Center Groningen according to Good Manufacturing Practice guidelines, as described previously (25), and administered intravenously with a predefined dose of 37 MBq (approximately 11 mg atezolizumab, < 1% of therapeutic dose). Imaging was performed seven days after injection at the optimal target-to-background ratio [25].

For the biodistribution of [^89^Zr]Zr-atezolizumab, the mean standardized uptake value (SUV_mean_) was measured in normal tissues using the ACCURATE tool [25, 28]. In patients with sequential [^89^Zr]Zr-atezolizumab PET/CT, the biodistribution of [^89^Zr]Zr-atezolizumab was also assessed after treatment with radium-223. To measure [^89^Zr]Zr-atezolizumab uptake in bone metastases, [^68^Ga]Ga-PSMA PET/CT was used as a reference standard to detect prostate cancer lesions (Fig. S2). Quantitative PET analyses are further described in the Supplemental Methods 1.2.

### Immune cells in blood and tumor biopsies

To measure circulating immune cells, blood samples were collected at baseline, before the second and fourth cycle of radium-223 (1–2 weeks prior to injection), at end of treatment, and at time of treatment failure. Tumor biopsies were obtained from ^68^Ga-PSMA positive bone lesions at baseline, and optionally after three cycles of radium-223 and at end of treatment. Detailed analyses are described in Supplemental Methods 1.3 and 1.4 and Tables S1 and S2.

### Statistical analysis

Statistical tests were chosen based on the format and normality distribution of the data and are described in the figure legends. All p-values were two-sided and *p* ≤ 0.05 was considered significant. Due to the exploratory nature of the study, no formal sample size was calculated and no correction for multiple testing was applied. For the key comparisons between responder and non-responders, the magnitude of the effect sizes was calculated using the matched-pairs rank-biserial correlation (r = (W^+^ - W^−^) / (W^+^ + W^−^)) for the Wilcoxon signed-rank test. The effect sizes for the Mann-Whitney U test were reported using the Hedges’ correction due to small sample size. In addition, to estimate the confidence interval of the p-values bootstrapping simulations were performed with a confidence interval of 95% (bias-corrected and accelerated) and a fixed number of samples (*N* = 5000). Statistical analyses were performed using SPSS (version 28.0.1.0).

## Results

### Baseline characteristics and clinical outcomes of included patients

Between February 2019 and January 2022, 28 consecutive patients were included, of whom 27 patients were evaluable (Fig. 3). Baseline characteristics were previously described (Tables S3 and S4) [27, 29]. In summary, patients had a mean age (± SD) of 70.1 ± 8.2 years, received a median of two prior treatment lines (IQR 1–2) and most (92%) patients had > 5 bone metastases on bone scintigraphy. Thirteen (48%) patients received six cycles of radium-223, while other patients discontinued treatment after 1–3 (*n* = 7) and 4–5 (*n* = 7) administrations. The most frequently reported reason for treatment discontinuation was progressive disease with (44%) or without (44%) new extra-osseous metastases. Two (7%) patients discontinued treatment due to hematological adverse events after four and five cycles of radium-223. For one patient, no imaging was performed at time of treatment failure because of clinical deterioration due to progressive disease. The median follow-up duration was 282 days (IQR 214–479, cut-off date 21st of October 2022). Overall, median FFS and OS were 22.9 weeks (IQR 15.0-25.9) and 48.0 weeks (IQR 27.0–91.0), respectively. Thirteen and 12 patients were categorized as good and poor responders, respectively. Two patients, who discontinued treatment due to adverse events, were excluded from the response analyses.

### PD-L1 expression in bone metastases on whole-body imaging decreases after radium-223

**Fig. 1:**
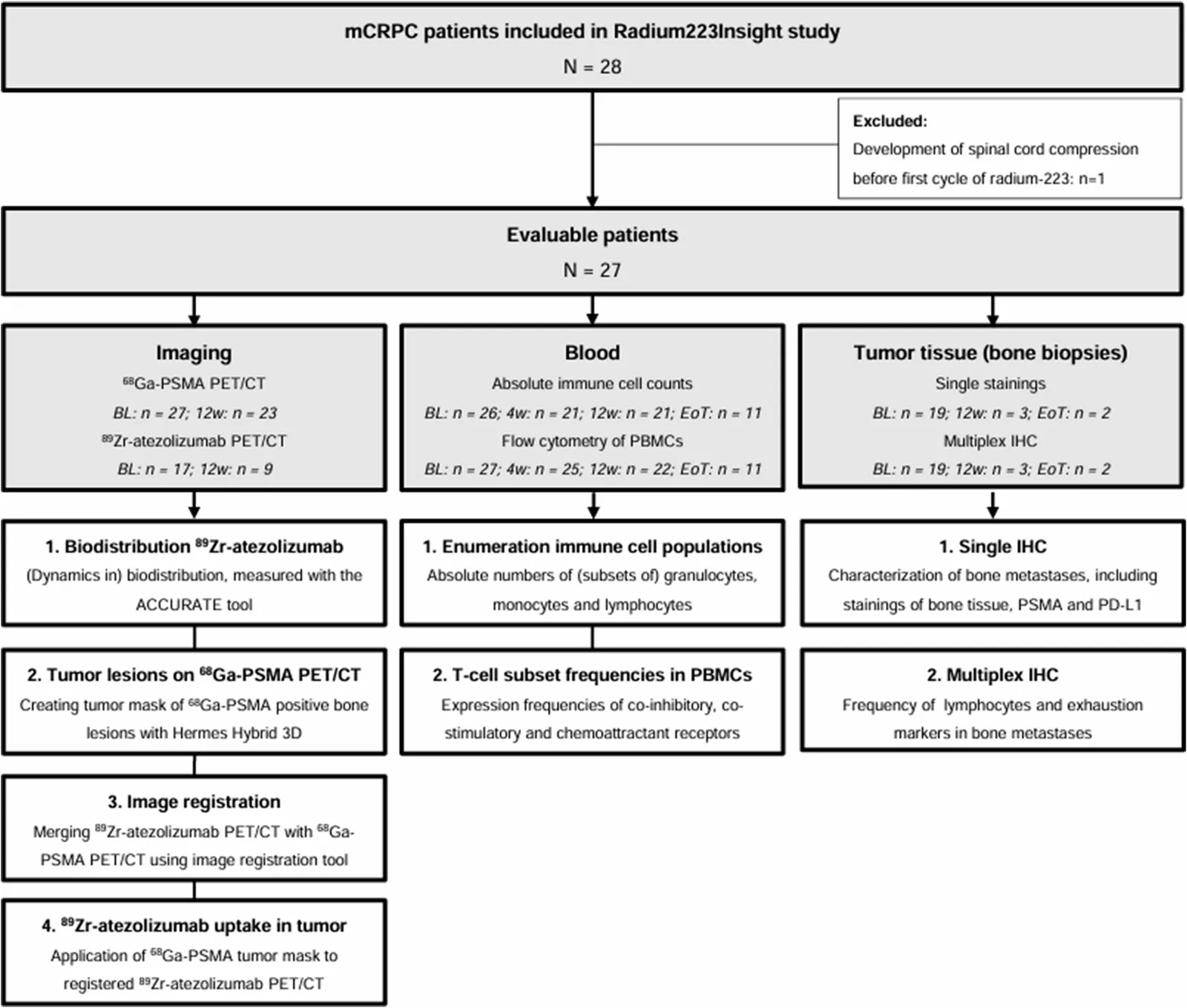
Flow chart of included patients and study procedures. mCRPC = metastatic castration-resistant prostate cancer, PSMA = prostate specific membrane antigen, BL = baseline, 12w = after three cycles of radium-223, EoT = end of treatment, IHC = immunohistochemistry, PBMC = peripheral blood mononuclear cells, PD-L1 = programmed cell death ligand 1

Paired PET images with [^68^Ga]Ga-PSMA and [^89^Zr]Zr-atezolizumab could be acquired at baseline (*n* = 17) and after three cycles of radium-223 (*n* = 9). One patient did not have bone metastases with ^68^Ga-PSMA uptake on follow-up images, which precluded accurate measurements of ^89^Zr-atezolizumab uptake. At baseline and after three cycles of radium-223, the biodistribution of [^89^Zr]Zr-atezolizumab was comparable in bone marrow (median SUV_mean_ 0.46 vs. 0.43) and bone cortex (median SUV_mean_ 0.46 vs. 0.43) (Fig. S3 and Table S5). The median number of ^68^Ga-PSMA positive lesions per patient was 11 (IQR 6–19) at baseline and 11 (IQR 5–23) after three cycles of radium-223 with a median total volume of 384 ml (IQR 113–908) and 416 ml (IQR 76-1449), respectively. After three cycles of radium-223, ^68^Ga-PSMA and ^89^Zr-atezolizumab uptake frequently decreased in the original bone metastases (Fig. 1) [27]. In addition, we observed lesions with stable ^68^Ga-PSMA uptake, but a decrease in ^89^Zr-atezolizumab uptake. After three cycles of radium-223, ^68^Ga-PSMA uptake often increased in the bone tissue adjacent to the original tumor lesions, but the ^89^Zr-atezolizumab uptake in these new ^68^Ga-PSMA positive lesions was lower than the ^89^Zr-atezolizumab uptake in the bone metastases at baseline.

**Fig. 2:**
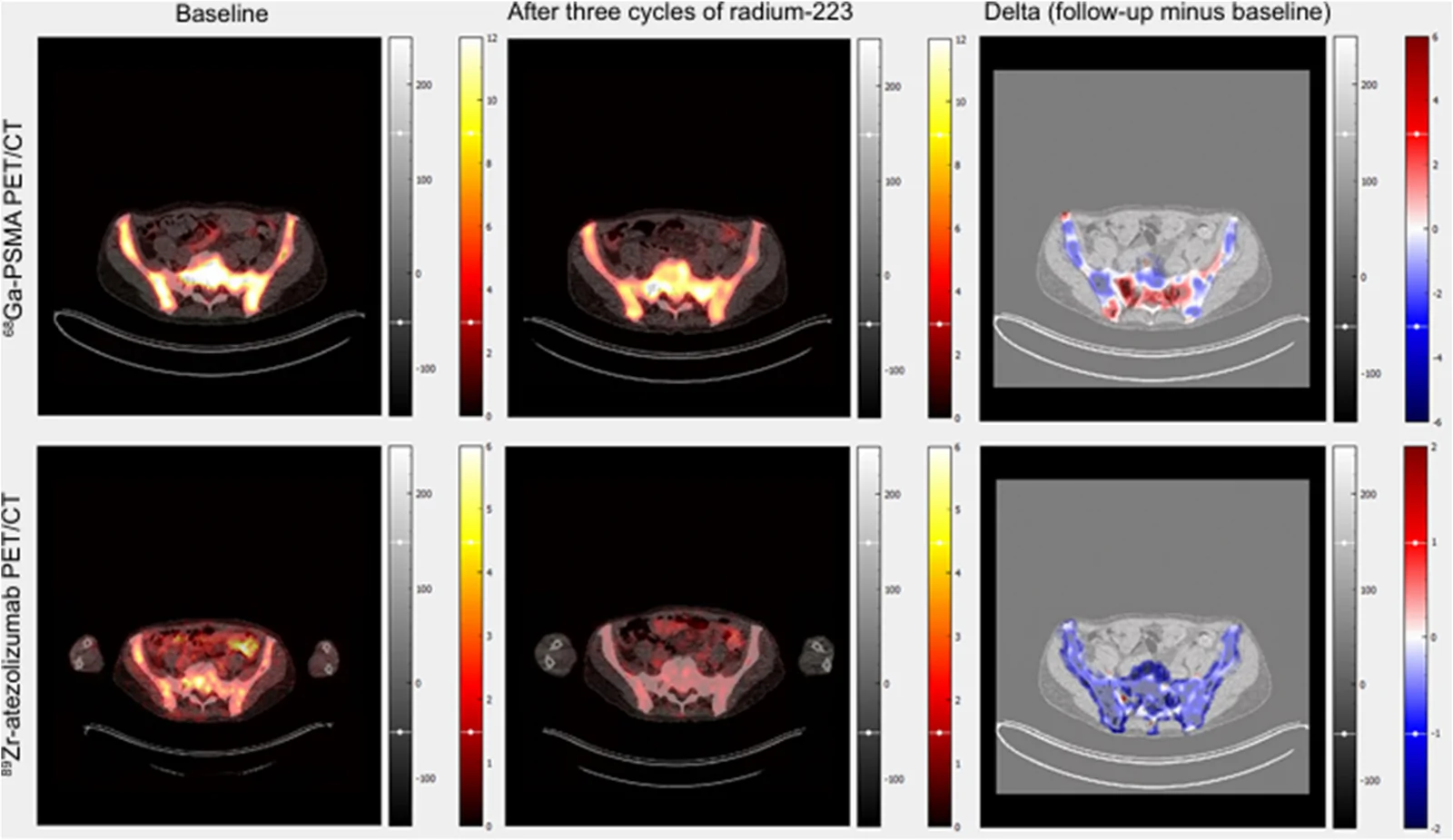
Example of 68Ga-PSMA PET/CT and ^89^Zr-atezolizumab PET/CT at baseline and after three cycles of radium-223 in one of the included patients. Scale bars represent Hounsfield Units of low-dose CT and standard uptake values (SUV) of 68Ga-PSMA and ^89^Zr-atezolizumab, respectively. Images on the right show the difference in SUV of 68Ga-PSMA and ^89^Zr-atezolizumab between baseline and after three cycles of radium-223 with red and blue representing increases and decreases in SUV over time, respectively

In the total volume of ^68^Ga-PSMA-positive bone lesions, the median SUV_mean_ of ^89^Zr-atezolizumab was 2.5 (IQR 0.9–3.4) at baseline and 0.9 (IQR 0.5–2.5) after three cycles of radium-223, which was higher than the physiological uptake in bone marrow at baseline (median 0.5, IQR 0.3–0.8, *p* < 0.001) and after three cycles of radium-223 (median 0.4, IQR 0.3–0.7, *p* = 0.069). Inter-patient and intra-patient variation in ^89^Zr-atezolizumab uptake in metastases was observed (Fig. 2a). After three cycles of radium-223, ^89^Zr-atezolizumab uptake in ^68^Ga-PSMA-positive bone lesions decreased with a median of 33% (*p* = 0.017, *r* = 0.75), while ^68^Ga-PSMA uptake was stable over time (*p* = 0.327, *r* = -0.25) (Fig. 2b-c). ^89^Zr-atezolizumab uptake in ^68^Ga-PSMA-positive bone lesions at baseline was comparable in good and poor responders (median SUV_mean_ 2.5 and 2.3, respectively), as well as the change in ^89^Zr-atezolizumab uptake over time (median change in SUV_mean_ -20% and − 44%, respectively) (Figs. 2d-e and S4).

### Immunohistochemistry shows varying PD-L1 expression in bone metastases

**Fig. 3:**
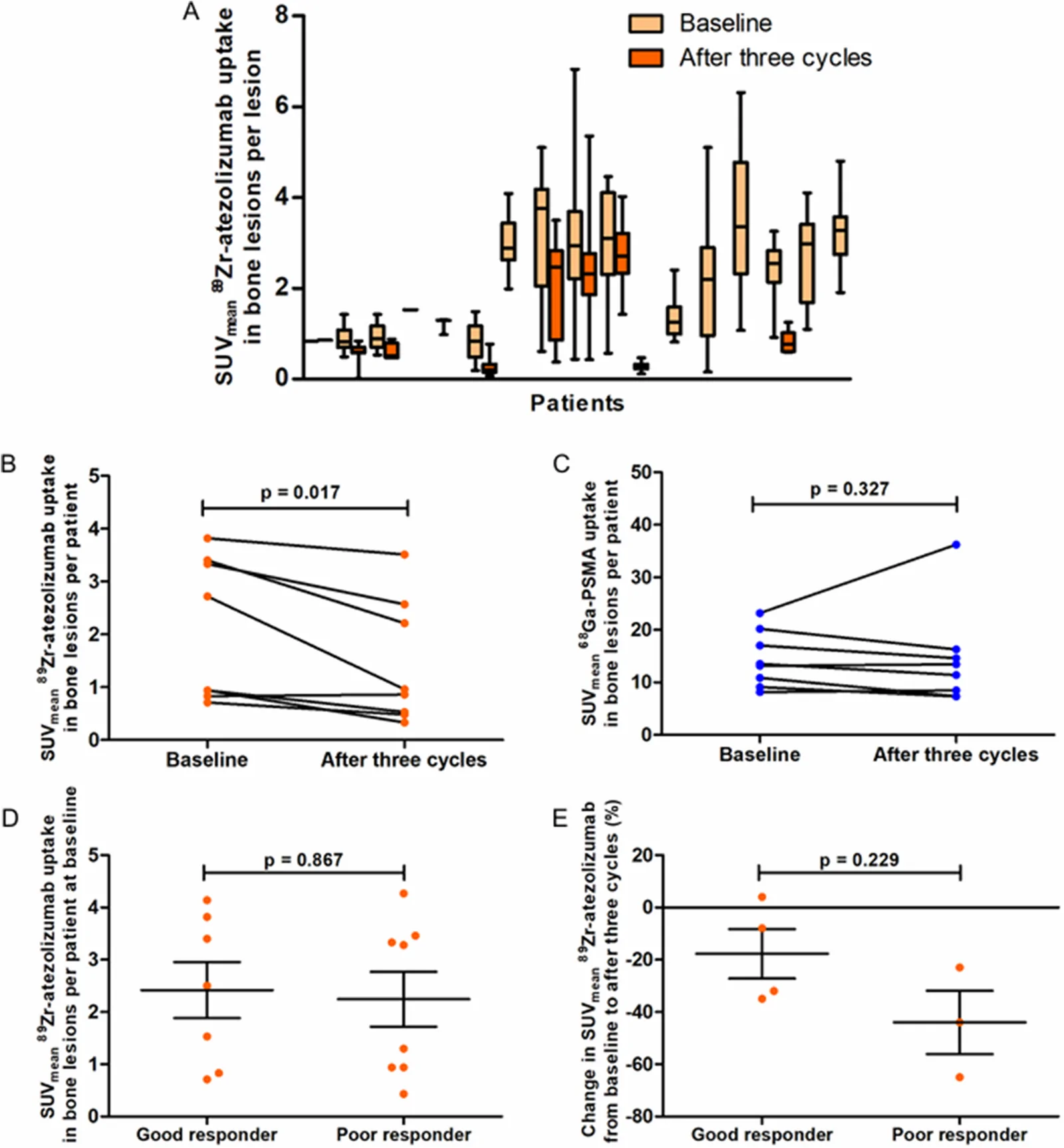
^89^Zr-atezolizumab uptake in 68Ga-PSMA-positive bone lesions. **A** SUVmean of ^89^Zr-atezolizumab in 68Ga-PSMA-positive bone lesions. Boxplots illustrate median, interquartile range and range of uptake in all lesions within each individual patient at baseline (n = 17) and after three cycles of radium-223 (n = 8). **B** Change in SUVmean of ^89^Zr-atezolizumab from baseline to after three cycles of radium-223 for patients with complete imaging at both time points (n = 8), Wilcoxon signed-rank test. **C** Change in SUVmean of 68Ga-PSMA from baseline to after three cycles of radium-223 for patients with complete imaging at both time points (n = 8), Wilcoxon signed-rank test. **D** Comparison of SUVmean of ^89^Zr-atezolizumab between good and poor responders to radium-223 (n = 7 and 8, respectively) at baseline, using a Mann-Whitney U test (p = 0.867, CI95% -1.366 – 1.727; r = 0.115). **E** Comparison of change in SUVmean of ^89^Zr-atezolizumab from baseline to after three cycles of radium-223 between good and poor responders (n = 4 and 3, respectively), using a Mann-Whitney U test (p = 0.229, CI95% 0.037 – 0.487; r = 1.11)

[^68^Ga]Ga-PSMA PET/CT guided bone biopsies were performed at baseline, after three cycles of radium-223 and at end of treatment in 25, 4 and 4 patients, respectively. Tumor cells were present in 19 (76%), 3 (75%) and 2 (50%) biopsies, respectively (Fig. 3). In general, bone biopsies at baseline (*n* = 19) consisted of cell-rich bone marrow, which included trabeculae, and immune and tumor cells. (Fig. S6). The majority of biopsies (*n* = 16) showed moderate to strong PSMA staining in 100% of the tumor cells, while three biopsies showed moderate PSMA staining in only 5%, 10% and 90% of the tumor cells, respectively (Fig. S6b). Mineralization of trabeculae varied widely from well to poorly calcified, reactive bone tissue (Fig. S6c). Osteocytes were present in the mineralized trabeculae, while few osteoblasts, which are the target of radium-223, were observed at the mineralization front of the trabeculae.

To investigate the origin of ^89^Zr-atezolizumab uptake on PET images, tumor biopsies from bone metastases were stained for PD-L1. PD-L1-expression was low in most biopsies and was mainly observed on tumor cells, while PD-L1-expression was also observed in non-tumor cells (Fig. S6d). After three cycles of radium-223, limited biopsies were available to measure changes in PD-L1 expression (Supplemental Results 2.1).

To further explore the composition of the immune microenvironment in mCRPC, the number of lymphocytes in tumor tissue and surrounding bone marrow was quantified in baseline bone biopsies (*n* = 19) (Figs. S6e, S7 and S8). Good responders to radium-223 had an abundance of TIM-3^+^ CD8^+^ T cells in bone metastases (Supplemental Results 2.2).

### Sequential biopsies show less lymphocytes in bone metastases after radium-223

Optional on- and post-treatment biopsies could be obtained in three patients. In two out of three patients, biopsies at baseline and after three cycles of radium-223 contained tumor cells. The post-treatment biopsy of one of these patients also contained tumor cells. In both patients, a decrease in the number of lymphocytes in tumor and bone marrow was observed after three cycles of radium-223 compared to baseline (Fig. S9c). In addition, one on-treatment biopsy revealed necrosis, which may have been caused by radiation effect (Fig. S10). In the corresponding post-treatment biopsy, an increase in the number of lymphocytes and development of woven bone tissue adjacent to the original trabeculae were observed (Figs. S9c and S10). Changes in lymphocyte subsets and expression of immune checkpoints over time were not comparable between the two patients (Figs. S8c and S9c).

### The frequency of circulating immune cells changes after radium-223

Changes in immune cell populations in blood were measured in available samples **(**Fig. 3**)** and a decrease in the number of circulating lymphocytes was measured after treatment with radium-223. The frequency of circulating PD-1 (CD279) expressing T cells did not differ between good and poor responders at baseline (CD4^+^: *p* = 0.462; CD8^+^: *p* = 0.723) (Fig. S11a and b). A significant increase in circulating TIM3^+^ T cells was observed after treatment with radium-223 in good responders (Supplemental Results 2.2 and 2.3).

## Discussion

Since irradiation might sensitize the microenvironment of metastatic prostate cancer for immunotherapy, we investigated changes in the micro- and macroenvironment of mCRPC in patients who were treated with radium-223. In bone metastases, ^89^Zr-atezolizumab uptake was low with high inter- and intra-patient heterogeneity, while PD-L1-expression was also low in most biopsies, reflecting the low immunogenicity of mCRPC. After three cycles of radium-223, the uptake of [^89^Zr]Zr-atezolizumab in ^68^Ga-PSMA-positive bone metastases further decreased and quantitative IHC and flow cytometry showed fewer lymphocytes in tumor tissue and blood, respectively.

The lower ^89^Zr-atezolizumab uptake after treatment with radium-223 might be caused by a decrease in PD-L1-expression per cell, or by a decrease in the absolute number of tumor and/or immune cells with PD-L1-expression. A decrease in the number of tumor cells is supported by the observed decrease in ^68^Ga-PSMA uptake in bone lesions after treatment with radium-223 [27]. Nevertheless, ^68^Ga-PSMA uptake can also increase in adjacent bone tissue after three cycles of radium-223, which is considered disease progression at the borderline of the initial bone lesions. With mainly PD-L1-expression on metastases, the dynamics of ^89^Zr-atezolizumab and ^68^Ga-PSMA uptake were expected to be comparable after treatment with radium-223. However, we observed a lower ^89^Zr-atezolizumab uptake in several bone lesions without a decrease in ^68^Ga-PSMA uptake. In addition, the increased ^68^Ga-PSMA uptake at the borders of the initial bone lesions during treatment was not accompanied by increased uptake of [^89^Zr]Zr-atezolizumab. Therefore, the changes in ^89^Zr-atezolizumab uptake could be caused by the observed treatment-induced drop in abundance of immune cells in the tumor microenvironment.

To investigate whether PD-L1 was mainly expressed on tumor or on immune cells in the bone metastases, PD-L1-expression in tumor biopsies was assessed using IHC [30]. When stained with SP-142 and 22C3, none of the biopsies showed PD-L1-expression. This may be caused by the low expression of PD-L1 in mCRPC, although underrepresentation cannot be excluded due to decalcification of samples prior to staining [31]. Nevertheless, multiplex IHC showed (low) expression of PD-L1 in bone metastases, which showed a trend with ^89^Zr-atezolizumab uptake and decreased after treatment with radium-223. Multiplex IHC showed that PD-L1 was expressed on both tumor and non-tumor cells .

Sequential tumor biopsies were compared to assess whether the decreased ^89^Zr-atezolizumab uptake was associated with a decrease in immune cell numbers. On-treatment biopsies showed a decrease in the number of lymphocytes compared to baseline biopsies, although this was based on paired biopsies from only two patients. However, the decrease in lymphocytes in tumor tissue was supported by the clear decreases in lymphocyte numbers in blood after radium-223, as observed in the current study and a previous study [32].

Some limitations should be considered when interpreting the results of this exploratory study. The observed decrease in ^89^Zr-atezolizumab uptake in bone metastases during treatment with radium-223 could have been confounded by several factors, including changes in vascularization over time or saturation of the targeted receptor using a radionuclide with a long half-life for sequential PET imaging. However, as the ^89^Zr-atezolizumab uptake in the bone cortex and bone marrow was stable over time, the decreased ^89^Zr-atezolizumab uptake in bone metastases is probably the result of treatment with radium-223. Finally, due to the rather high number of invasive procedures in this study, sequential biopsies were only feasible in a limited number of patients, and the quality of the bone biopsies was moderate. Overall, bone histology showed disrupted trabecular connections and loss of trabeculae, replaced by bone marrow and nonmineralized woven bone in patients with mCRPC during radium-223 treatment.

Overall, our findings did not show any increase in PD-L1-expression after treatment with radium-223, which indicates that radium-223 cannot sensitize prostate cancer to PD-L1 inhibition. This is confirmed by a phase Ib and two phase II studies that showed no benefit of radium-223 when added to atezolizumab or pembrolizumab or enzalutamide in patients with mCRPC [33–35]. In contrast to our findings, a retrospective study showed a correlation between different tumor infiltrating lymphocytes and therapeutic efficacy in patients with prostate cancer who had salvage radiotherapy after biochemical recurrence [36]. Another preclinical study also suggested an increased therapeutic efficacy by combining radium-223 with immune checkpoint inhibitors [22]. Although possible synergistic effects between radiotherapy or alpha emitting radium-223 and immune checkpoint inhibition were shown in these studies, the design and patient population were different from those in the current study. Nevertheless, if radium-223, or even irradiation with either alpha or beta emitters in general, results in extensive cell death of immune cells in the tumor microenvironment, the immunogenic effect of irradiation may be hampered by the absolute decrease in the number of immune cells. Therefore, new combination strategies combining a radionuclide with a lower linear energy transfer in a lower dose, such as ^177^Lu-PSMA, could be considered to induce immunogenicity [36–39].

## Conclusion

Treatment of mCRPC bone metastases with radium-223 results in a significant decrease in in vivo PD-L1-expression on PET imaging. These findings, in combination with a decreased number of immune cells in tumor biopsies and blood, indicate that radium-223 is not able to sensitize prostate cancer to PD-L1 inhibition and alters the immune microenvironment, thereby potentially reducing the efficacy of PD-(L)1 inhibitors.

## Supplementary Information

Supplementary Material 1.

Supplementary Material 2.

Supplementary Material 3.

## Data Availability

The data that support the findings of this study are available upon reasonable request from the corresponding author.
