# Supplementary material for: Radium-223 decreases PD-L1 expression in bone metastases: A study with [89Zr]Zr-atezolizumab PET and immunohistological correlation
Source: Eur J Nucl Med Mol Imaging. 2026 Sep 12;53(13):8558–67. doi: 10.1007/s00259-026-08175-9 (PMC13727859; doi:10.1007/s00259-026-08175-9)
Supplement: Supplementary file 1 [file 259_2026_8175_MOESM1_ESM.pdf]

Radium223Insight

A response measurement study in metastatic castration-resistant prostate cancer patients, treated with radium-223.

Version 3.0

PROTOCOL TITLE 'Response measurement study in metastatic castration-resistant prostate cancer patients, treated with radium-223, to improve early response evaluation and understand the radium-223 induced immune response.'

**Protocol ID**	NL66323.078.18
**Short title**	Radium223Insight
**EudraCT number**	Not applicable
**Version**	3.0
**Date**	11-12-2019
**Principal investigator**	**A.A.M van der Veldt, MD PhD**<br>Erasmus Medical Center<br>Department of Medical Oncology<br>Dr. Molewaterplein 40, 3015 GD Rotterdam<br>The Netherlands<br>Tel: +31 (0)6 18 84 27 60<br>E-mail: a.vanderveldt@erasmusmc.nl
**Writing committee**	A.C. de Jong, MD<br>D.G.J. Robbrecht, MD<br>M.P.J.K. Lolkema, MD PhD<br>Prof. dr. R. de Wit, MD<br>A.A.M. van der Veldt, MD PhD
**Local investigator(s)**	**N. Mehra, MD PhD**<br>Radboud University Medical Center<br>Geert Grooteplein Zuid 10, 6525 GA Nijmegen<br>The Netherlands<br>Tel: +31 (0)24 361 03 53<br>E-mail: niven.mehra@radboudumc.nl<br><br>**A.P. Hamberg, MD PhD**<br>Franciscus Gasthuis & Vlietland<br>Department of Internal Medicine<br>Kleiweg 500, 3045 PM, Rotterdam<br>The Netherlands

	Tel: +31 (0)10 461 61 61<br>E-mail: p.hamberg@franciscus.nl
**Coordinating investigator**	**A.C. de Jong, MD**<br>Erasmus Medical Center<br>Department of Medical Oncology<br>Dr. Molewaterplein 40, 3015 GD Rotterdam<br>Tel: +31 (0)6 18 19 77 82<br>Fax: +31 (0)10 704 10 03<br>E-mail: a.c.dejong@erasmusmc.nl
**Sponsor**	**Erasmus Medical Center Cancer Institute**<br>Department of Medical Oncology
**Subsidising party**	**Bayer**
**Independent experts**	**Erasmus MC Rotterdam**<br>M. Jongen-Lavrencic, MD PhD<br>Department of Hematology<br>Dr. Molewaterplein 40, 3015 GD Rotterdam<br>The Netherlands<br>Tel: +31 (0)10 704 13 67<br>E-mail: m.lavrencic@erasmusmc.nl<br><br>**Franciscus Gasthuis en Vlietland**<br>Dr. J.C.C.M in 't Veen, MD PhD<br>Department of Pulmonary Disease<br>Kleiweg 500<br>3045 PM, Rotterdam<br>The Netherlands<br>Tel: +31 10 461 76 32<br>E-mail: h.intveen@franciscus.nl<br><br>**Radboud UMC Nijmegen**<br>Dr. A.J. Croockewit, MD PhD<br>Department of Hematology<br>Geert Grooteplein Zuid 10, 6525 GA Nijmegen<br>The Netherlands

	Tel: +31 (0)24 361 47 62<br>E-mail: sandra.croockewit@radboudumc.nl
**Laboratory sites**	**Erasmus MC Rotterdam**<br>Department of Medical Oncology<br>Laboratory of Translational Cancer Genomics and Proteomics<br>*Prof. dr. J.W.M. Martens*<br><br>Laboratory of Tumor Immunology<br>*Dr. R. Debets*<br><br>Department of Radiology and Nuclear Medicine<br>*Dr. T. Brabander*<br><br>**Radboud UMC Nijmegen**<br>Department of Medical Oncology<br>Laboratory of Tumor Immunology<br>*Prof. dr. I.J.M. de Vries*<br><br>**UMC Groningen**<br>Department of Medical Oncology<br>*Prof. dr. E.G.E. de Vries*<br><br>Department of Radiology and Nuclear Medicine<br>*Dr. A.H. Brouwers*<br>*Dr. A. Jorritsma*
**Pharmacy**	**Erasmus MC Rotterdam**<br>*Dr. S.L.W. Koolen*

PROTOCOL SIGNATURE SHEET

Name	Signature	Date
**Sponsor or legal representative:**<br>Erasmus Medical Center<br>Department of Medical Oncology<br><br>**Head of Department:**<br>S. Sleijfer, MD PhD		12/12/19
**Principal Investigator:**<br>A.A.M. van der Veldt, MD PhD		11-12-2019

TABLE OF CONTENTS

1. INTRODUCTION AND RATIONALE 13
1.1 Prostate cancer 13
1.1.1 Treatment of metastatic prostate cancer 13
1.2 Radium-223....13
1.2.1 ALSYMPCA trial followed by FDA approval....13
1.2.2 Mechanism of action 14
1.3 Response evaluation during radium-223 treatment 14
1.3.1 Traditional response biomarkers 14
1.3.2 Identification of new response biomarkers....15
1.4 Understanding the radium-223 induced immune response....17
1.4.1 Immunotherapy in prostate cancer 17
1.4.2 Abscopal effect and immunogenic cell death....18
1.4.3 Exploring the radium-223 induced immune response 18
1.5 Radium223Insight 20
2. OBJECTIVES....21
2.1 Primary objectives....21
2.2 Secondary objectives 21
3. STUDY DESIGN 22
3.1 Study treatment....22
3.2 Main study procedures 22
3.2.1 Identification of response biomarkers 22
3.2.2 Immune profiling....23
3.2.3 Molecular profiling 24
4. STUDY POPULATION 25
4.1 Population (base) 25
4.2 Inclusion criteria 25
4.3 Exclusion criteria 25
4.4 Sample size calculation....26
5. TREATMENT OF SUBJECTS 27
5.1 Investigational product 27
5.2 Use of co-intervention 27
5.3 Escape medication 27
6. INVESTIGATIONAL PRODUCT....28
7. NON-INVESTIGATIONAL PRODUCT....29
7.1 Name and description of non-investigational product(s) 29
7.2 Dosages, dosage modifications and method of administration 29
7.3 Preparation and labelling of Non Investigational Medicinal Product....29
8. METHODS 31
8.1 Study parameters/endpoints....31
8.1.1 Main study endpoints 31
8.1.2 Secondary study endpoints 32
8.2 Randomisation, blinding and treatment allocation 33

8.3 Study procedures 33
8.3.1 Baseline screening 34
8.3.2 At baseline, prior to administration of radium-223 34
8.3.3 Before the second cycle of radium-223 34
8.3.4 Before the third cycle of radium-223 35
8.3.5 Before the fourth cycle of radium-223 35
8.3.6 Before the fifth and sixth cycle of radium-223 35
8.3.7 Four weeks after the last radium-223 injection 35
8.3.8 Follow-up 36
8.3.9 Progression of disease 36
8.4 Withdrawal of individual subjects 36
8.4.1 Specific criteria for withdrawal 37
8.5 Replacement of individual subjects after withdrawal 37
8.6 Follow-up of subjects withdrawn from treatment 37
8.7 Premature termination of the study 37
9. SAFETY REPORTING 38
9.1 Temporary halt for reasons of subject safety 38
9.2 AEs, SAEs and SUSARs 38
9.2.1 Adverse events (AEs) 38
9.2.2 Serious adverse events (SAEs) 38
9.2.3 Suspected unexpected serious adverse reactions (SUSARs) 39
9.3 Annual safety report 39
9.4 Follow-up of adverse events 40
9.5 Data Safety Monitoring Board (DSMB) / Safety Committee 40
10. STATISTICAL ANALYSIS 41
11. ETHICAL CONSIDERATIONS 42
11.1 Regulation statement 42
11.2 Recruitment and consent 42
11.3 Objection by minors or incapacitated subjects 42
11.4 Compensation for injury 42
11.5 Incentives 43
12. ADMINISTRATIVE ASPECTS, MONITORING AND PUBLICATION 44
12.1 Handling and storage of data and documents 44
12.2 Monitoring and Quality Assurance 44
12.3 Amendments 44
12.4 Annual progress report 45
12.5 Temporary halt and (prematurely) end of study report 45
12.6 Public disclosure and publication policy 45
13. STRUCTURED RISK ANALYSIS 46
13.1 Potential issues of concern 46
13.2 Synthesis 46
14. REFERENCES 48

LIST OF ABBREVIATIONS AND RELEVANT DEFINITIONS

**ABR**	ABR form, General Assessment and Registration form, is the application form that is required for submission to the accredited Ethics Committee (In Dutch, ABR = Algemene Beoordeling en Registratie)
**ADT**	Androgen Deprivation Therapy
**AE**	Adverse Event
**APC**	Antigen Presenting Cell
**AR**	Adverse Reaction
**AR**	Androgen Receptor
**CA**	Competent Authority
**CALR**	Endoplasmic Reticulum Chaperone Calreticuline
**CCMO**	Central Committee on Research Involving Human Subjects; in Dutch: Centrale Commissie Mensgebonden Onderzoek
**cfDNA**	Circulating free DNA
**CPCT**	Center for Personalized Cancer Treatment
**CRPC**	Castration Resistant Prostate Cancer
**CT**	Computed Tomography
**CTC**	Circulating tumor cell
**ctDNA**	Circulating tumor-derived DNA
**CTLA-4**	Cytotoxic T lymphocyte Associated Protein 4
**CV**	Curriculum Vitae
**DAMP**	Danger Associated Molecular Patterns
**DSMB**	Data Safety Monitoring Board
**ECOG**	Eastern Cooperative Oncology Group
**EU**	European Union
**EudraCT**	European drug regulatory affairs Clinical Trials
**[^{18}F]PSMA**	^{18}Fluor Prostate Specific Membrane Antigen
**FFS**	Failure free survival
**FFPE**	Formaline Fixed Paraffin Embedded
**[^{68}Ga]PSMA**	^{68}Gallium Prostate Specific Membrane Antigen
**GCP**	Good Clinical Practice
**HMGB1**	High Mobility Group Box 1
**IB**	Investigator’s Brochure
**IC**	Informed Consent
**ICD**	Immunogenic Cell Death
**IMP**	Investigational Medicinal Product

**IMPD**	Investigational Medicinal Product Dossier
**LDH**	Lactate Dehydrogenase
**mCRPC**	Metastatic Castration Resistant Prostate Cancer
**METC**	Medical research ethics committee (MREC); in Dutch: medisch ethische toetsing commissie (METC)
**NGS**	Next Generation Sequencing
**PBMC**	Peripheral Blood Mononuclear Cell
**PD-1**	Programmed Cell Death 1
**PD-L1**	Programmed Cell Death Ligand 1
**PET**	Positron Emission Tomography
**PFS**	Progression Free Survival
**PSA**	Prostate Specific Antigen
**PSMA**	Prostate Specific Membrane Antigen
**OS**	Overall survival
**RCT**	Randomized Controlled Trial
**SAE**	Serious Adverse Event
**SPC**	Summary of Product Characteristics (in Dutch: officiële productinfomatie IB1-tekst)
**Sponsor**	The sponsor is the party that commissions the organisation or performance of the research, for example a pharmaceutical
**SRE**	Skeletal Related Event
**SUSAR**	Suspected Unexpected Serious Adverse Reaction
**TAA**	Tumor Associated Antigen
**tALP**	Total Alkalic Phosphatase
**TCR**	T Cell Receptor
**ULN**	Upper Limit of Normal
**Wbp**	Personal Data Protection Act (in Dutch: Wet Bescherming Persoonsgevens)
**WGS**	Whole Genome Sequencing
**WMO**	Medical Research Involving Human Subjects Act (in Dutch: Wet Medisch-wetenschappelijk Onderzoek met Mensen
	company, academic hospital, scientific organisation or investigator. A party that provides funding for a study but does not commission it is not regarded as the sponsor, but referred to as a subsidising party.
**^{89}Zr**	^{89}Zirconium

SUMMARY

Rationale: Based on the survival results of a randomized phase 3 trial, the European Medicines Agency (EMA) and Committee for the Assessment of Oncological Medicines (‘Commissie BOM’) have approved radium-223 for the treatment of metastatic castration resistant prostate cancer (mCRPC) with bone metastases only. As traditional parameters, including PSA, fail in (early) response evaluation, other parameters are needed to guide treatment planning in mCRPC patients. As the efficacy of immunotherapy is limited in mCRPC and radium-223 may initiate an immune response by activation of CD8 T lymphocytes, further understanding is required for the rational development of combination strategies, including those with radium-223.

Objective:
The primary objectives are:
1. To identify biomarkers for early identification of clinical benefit from radium-223 treatment in mCRPC patients.
2. To better understand immune response during radium-223 treatment in mCRPC patients.
The secondary objective is:
1. To characterize the mutational and immunological profile of pre-treatment tumor genomes of mCRPC patients in relation to clinical benefit.

Study design: This is a prospective, translational, multicenter, hypothesis-generating study with an exploratory design.

Study population: The study population consists of men with mCRPC with bone metastases only, who are eligible for radium-223 treatment.

Intervention: Patients will be treated with radium-223 according to standard of care. Prior and during treatment, multi-parametric biomarkers will be collected. These biomarkers are obtained from blood samples, imaging and tissue.

<u>Blood samples:</u>
- Conventional markers (PSA, ALP, LDH)
- Circulating tumor cells
- Circulating tumor derived DNA
- Immune cells and markers

Imaging:

- Conventional imaging (CT and bone scintigraphy)
- [^{68}Ga]PSMA PET-CT
- [^{89}Zr]atezolizumab PET-CT

Tissue (bone biopsy):

- Whole genome sequencing, focusing on mutational and immune profiling data.
- In situ multiplex immune fluorescence of FFPE using automated quantitative pathology imaging.

Main study endpoints:

1. Exploratory analysis of multiple biomarkers in relation to failure-free survival, defined as time to next line of treatment, best supportive care or death. Next line of treatment or best supportive care will be started upon clinical, biochemical and/or radiological signs of progression according to the PCWG3 criteria.

2. Exploratory analysis of the immune response during radium-223 treatment in relation to failure-free survival as defined above.

Nature and extent of the burden and risks associated with participation, benefit and group relatedness: Participation in this study requires additional blood draws, imaging, and a pre-treatment bone biopsy.

Total needed blood volume varies between 30-80 ml per blood draw (including standard laboratory examinations). Blood draws will take place before each cycle (every four weeks), during follow-up and upon progression in combination with regular blood draws. Additionally, a limited amount of blood will be drawn to measure the radioactivity of the tracers in blood, which is necessary for good imaging analysis. The risk of this limited amount of blood being drawn is minimal.

Additionally, patients will undergo four imaging modalities during treatment: three bone scintigraphies, three CT scans of the thorax and abdomen, CT-guidance during the bone biopsy, three [^{68}Ga]PSMA PET-CT scans and two [^{89}Zr]atezolizumab PET-CT scans. The bone scintigraphy and the CT-thorax/abdomen at baseline and at the end of treatment are standard of care. The remaining scans will give an additional radiation burden of 80 mSv, divided over 24 weeks. During follow-up, CT scans and bone scintigraphies will be performed according to

standard of care. At disease progression, an additional [^{68}Ga]PSMA PET-CT will be performed, which accounts for 6 mSv. Based on the ALSYMPCA trial, the patient population in this study will have a median age of 71 and a median survival of approximately 15 months. Although the absolute additional radiation burden is considerable, this is only about 3% of the estimated effective dose of the radium-223 treatment, which accounts for approximately 3000 mSv. Considering the age of the patients, the prognosis, the relative small additional radiation burden on top of the standard radium-223 treatment and the substantial benefit of this study for future treatment of prostate cancer patients, the radiation burden of the imaging protocol is considered justified.

The risk of the bone biopsy is considered low (1). All safety measures and procedures will be performed according to local guidelines.

1. INTRODUCTION AND RATIONALE

1.1 Prostate cancer

With approximately 11,000 men diagnosed in 2016, prostate cancer is the most common non-skin malignancy in men in the Netherlands (2). Although most of the patients (70%) present with local disease at diagnosis, 20-40% develops recurrent and/or metastatic disease within 10 years after primary treatment, resulting in the death of approximately 2500 men each year (3, 4). Bone metastases, which are found in more than 80% of the patients, who die from prostate cancer, are a major problem, since they often cause pain and skeletal related events (SRE) (2, 5, 6). SREs are associated with increased mortality (7).

1.1.1 Treatment of metastatic prostate cancer

Initial treatment of metastatic prostate cancer is based on the depletion of testosterone through surgical or chemical castration, called androgen deprivation therapy (ADT), which can be combined with docetaxel (8-10). However, within approximately 14-20 months the cancer will progress and the disease enters a new stage, called castration resistant prostate cancer (CRPC) (11). For patients with mCRPC, several treatment options are currently available including chemotherapy, androgen receptor (AR) – targeted therapy, and radium-223. Patients, who are fit enough to receive chemotherapy, can be treated with docetaxel (12). When patients had benefit from ADT for ≥ 12months before developing CRPC and experience mild or no symptoms from their disease, treatment with the AR – targeted therapy abiraterone or enzalutamide can be considered as first line therapy (13, 14). Patients with symptomatic bone metastases without visceral metastases are eligible for treatment with radium-223 (15). In case of progressive disease during first-line therapy, patients can be treated in second-line with abiraterone, enzalutamide, radium-223, docetaxel or cabazitaxel, a next-generation tubulin-stabilizing taxane (13, 15-17). For second-line treatment, no head-to-head comparisons are available and predictive factors to select CRPC patients for a specific second-line treatment are urgently needed.

1.2 Radium-223

1.2.1 ALSYMPCA trial followed by FDA approval

In the ALSYMPCA trial, 921 symptomatic mCRPC patients who had received, were not eligible to receive or declined docetaxel were randomized to receive six injections of the alpha-emitting radiopharmaceutical radium-223 every four weeks or matching placebo (15). Treatment with radium-223 improved overall survival (OS) compared to placebo (median, 14.9 months *vs.* 11.3 months; hazard ratio, 0.70; 95% CI, 0.58 to 0.83; P<0.001) and significantly prolonged

the time to the first symptomatic skeletal event (median, 15.6 months *vs.* 9.8 months; hazard ratio, 0.66; 95% CI, 0.52 to 0.83; P<0.001). In addition, the overall incidence of adverse events was lower in the radium-223 group than in the placebo group (93% *vs.* 96%) with no clinically meaningful differences in the frequency of hematologic adverse events. Based on these results, the Food and Drug Administration (FDA) approved radium-223-dichloride in 2013 for mCRPC patients with symptomatic bone metastases without visceral metastases.

1.2.2 Mechanism of action

As mentioned above, bone metastases have serious clinical implications. Although external beam irradiation can be used to relieve pain of isolated lesions, it is not tumor specific. Radiation of large fields harms the surrounding tissue, thereby regularly resulting in bone marrow suppression and gastrointestinal toxicity (18). To treat multifocal pain caused by bone metastases, treatment with bone-seeking radiopharmaceuticals can be considered. By imitating calcium in bone forming complexes with the mineral hydroxyapatite, these bone-seeking radiopharmaceuticals target areas with high bone turnover, like osteoblastic bone metastases. For the treatment of mCRPC, the clinically most relevant bone-seeking radiopharmaceuticals include the alpha and beta emitting particles. Most of these agents, including strontium-89, samarium-153, rhenium-186, and rhenium-188 emit beta particles, whereas radium-223 is an alpha-emitter (19). In contrast to beta-emitting radiopharmaceuticals, which have a range of 2.5-10 mm, the alpha-particles of radium-223 emit a relatively high amount of energy within a very short range of < 100 um (19). This results in a local effect with double-stranded DNA breaks in the nearest cells only and a minimal toxic effect on the surrounding tissue, like the bone marrow (20). In mCRPC patients, all mentioned radiopharmaceuticals initiate at least a 50% pain response (19). Radium-223 is the only agent that has been proven to increase OS (15).

1.3 Response evaluation during radium-223 treatment

Adequate response evaluation during radium-223 may help to understand the efficacy and subsequently improve treatment planning. However, traditional response parameters for prostate cancer, e.g. PSA, are not useful. Therefore, the rational introduction of new treatment strategies containing radium-223 are challenging, underscoring the need for new biomarkers.

1.3.1 Traditional response biomarkers

Blood based biomarkers

As blood based parameters are minimally invasive, they enable frequent and sequential measurements for response evaluation. In clinical practice, prostate specific antigen (PSA) is commonly assessed to evaluate prostate cancer. In the metastatic setting, PSA is less useful,

as PSA levels are regulated by an androgen-dependent promoter and consequently affected by androgen deprivation therapy (21, 22). In addition, PSA levels are not predictive during radium-223, as illustrated by an exploratory analysis of the ALSYMPCA trial (23). In this analysis, a decline of two other biomarkers, total alkaline phosphatase (tALP) and lactate dehydrogenase (LDH), at 12 weeks did correlate with longer OS, but tALP and LDH did not meet statistical surrogacy requirements for OS. (23).

<u>Imaging based biomarkers</u>

As prostate cancer is mainly localized in the bone, conventional anatomical imaging techniques, such as computed tomography (CT) and magnetic resonance imaging (MRI), are not sufficient for response evaluation during radium-223 (24). In addition, ^{18}fluorodeoxyglucose-PET (^{18}FDG-PET), a functional imaging technique, is not useful either, as prostate cancer is not avid to ^{18}FDG (25). Although ^{99m}Technetium (^{99m}Tc) bone scintigraphy is currently the standard imaging modality for bone metastases in prostate cancer (26), its use for early response evaluation is limited because of the potential false positive flare after start of treatment (26).

<u>Reference standard</u>

As most clinical outcome measures are not useful as a reference for early response evaluation during radium-233, the introduction and development of new response markers is challenging. Clinical outcome measures, such as progression-free survival (PFS) and failure-free survival (FFS), are not completely applicable, as PCWG3 criteria for disease progression appear not sufficient during radium-223. On the other hand, OS is a late outcome measure that is affected by the therapies that are given besides radium-223. Therefore, time to next line of treatment is considered as the best reflection of clinical benefit during radium-223, since this is not influenced by other systemic therapy and is initiated upon signs and/or symptoms of progressive disease.

1.3.2 Identification of new response biomarkers

<u>Imaging: PSMA PET-CT</u>

As prostate cancer is not ^{18}FDG avid, other more prostate-specific PET tracers have been developed. An important target is prostate specific membrane antigen (PSMA), a cell surface protein, which is highly expressed in epithelial prostate cells and prostate cancer cells (27, 28). Since its introduction in 2012, the [^{68}Ga]PSMA PET-CT is an emerging diagnostic tool with high sensitivity and specificity for the staging of prostate cancer (29-31). In addition, [^{68}Ga]PSMA PET-CT is a promising tool for response evaluation of docetaxel and external

beam radiotherapy (32, 33). Since [^{68}Ga]PSMA PET-CT has a higher diagnostic accuracy (sensitivity 98.8–99.0% and specificity 98.9–100%) than the traditional bone scintigraphy (sensitivity 82.4–86.6% and specificity 91.6–97.9%) for bone metastases, [^{68}Ga]PSMA PET-CT is promising for response evaluation during radium-223 (34). Besides [^{68}Ga]PSMA, other PSMA-tracers, including [^{18}F]PSMA-1007, have been developed to optimize image quality. As [^{18}F]PSMA-1007 PET-CT shows a reduced urinary excretion, thereby improving image quality of the prostate region, [^{18}F]PSMA-1007 is increasingly used and currently replacing [^{68}Ga]PSMA in the clinic (35).

Tumor biopsies

For the development of more personalized treatment strategies in prostate cancer, information on deregulated genes or signaling pathways, that are potentially targetable by drugs, is essential. The mutational profile of tumors and the germline DNA background variation can be obtained by whole genome sequencing (WGS) of tumor tissue and peripheral blood, respectively, enabling extensive analysis of mutational patterns (e.g. BRCA like mutagenesis) and mutations in genes involved in DNA repair pathways (1).

Liquid biopsies: circulating tumor cells (CTCs)

In approximately 80% of mCRPC patients, circulating tumor cells (CTCs) are found in the peripheral circulation (36). These CTCs, which detach from the primary and metastatic tumor sites, can be collected via a regular blood draw, a so-called liquid biopsy, and are isolated with the CellSearch system. Compared with traditional biopsies, CTCs are considered to better reflect intra- and inter-tumor heterogeneity, as CTCs do not origin from one lesion (36). Although present at relatively low frequencies, enumeration and characterization of CTCs have a prognostic and predictive value. In mCRPC patients commencing a new line of chemotherapy, the presence of <5 CTCs in 7,5ml of blood at baseline predicts a favorable OS, while five or more CTCs at baseline are associated with an unfavorable prognosis (37). Also during chemotherapy in mCRPC patients, the CTC count is predictive for OS. Already two to five weeks after start of the treatment, a conversion in CTC count predicts an improvement or deterioration of the prognosis (37). Based on these results, the FDA approved the enumeration of CTCs for the evaluation of mCRPC. Although CTCs may be useful for response evaluation during radium-233, only one exploratory study studied CTCs in 19 mCRPC patients treated with radium-223 (38). Therefore, further studies are needed to evaluate the predictive value of CTCs in mCRPC patients treated with radium-223.

Liquid biopsies: circulating tumor DNA (ctDNA)

Besides CTCs, small fragments of DNA from apoptotic and necrotic tumor cells can be detected in blood (39-41). In this circulating tumor derived DNA (ctDNA), structural variants (SVs) can be detected. These SVs are genomic rearrangements of ≥ 50 base pairs, including inversions, insertions, mobile-element transpositions, translocations, duplications, amplifications and deletions. Quantification of SVs, using PCR based techniques, varies upon treatment or disease progression (42, 43). By measuring the quantity of an SV over time we aim to evaluate treatment response to radium-223.

1.4 Understanding the radium-223 induced immune response

The interaction between tumor and immune system plays an important role in the development of cancer. Although for a long time the immune system was considered to be an ever-alert surveillance system, it has become clear that some tumor cells are able to surpass this system (44). One way to escape the immune system is via the expression of programmed cell death ligand 1 (PD-L1) on the surface of tumor cells. Binding of PD-L1 to the PD-1 receptor on T cells inhibits activation of the immune system, thereby inhibiting the anti-tumor response (45).

Immunotherapy aims to induce an anti-tumor response by activating the patient's own immune system. By inhibiting immune checkpoints with monoclonal antibodies that block the function of inhibitory receptors on T cells, inhibition of T cells is released. Treatment with immune checkpoint inhibitors was shown to be effective in a variety of solid tumors. Thus far, antibodies against programmed cell death 1 (PD-1) (e.g. nivolumab and pembrolizumab), PD-L1 (e.g. atezolizumab) and cytotoxic T-lymphocyte-associated protein 4 (CTLA-4) (e.g. ipilimumab) have been introduced in the clinic (46).

1.4.1 Immunotherapy in prostate cancer

For the treatment of mCRPC, autologous cell-based vaccination therapy sipuleucel-T is the only approved immunotherapeutic agent. For sipuleucel-T, autologous peripheral-blood mononuclear cells (PBMCs), including antigen-presenting cells (APCs), are activated *ex vivo* with PA2024, a recombinant fusion protein. PA2024 consists of a prostate antigen and a granulocyte-macrophage colony stimulating factor that serves as an immune-cell activator. In mCRPC patients, sipuleucel-T improved the median OS with 4.1 months as compared to placebo (47).

The efficacy of immune checkpoint inhibitors in mCRPC appears limited. Although ipilimumab did not improve OS as compared to placebo, there is a trend towards an improved progression free survival (PFS), suggesting some efficacy (48, 49). Furthermore, an early phase 2 study showed robust responses when pembrolizumab was added to enzalutamide in patients with

progressive disease during previous treatment with enzalutamide (50). In this study, pre-treatment biopsies of the responders showed presence of CD3+, CD8+ and CD163+ leukocyte infiltrates and PD-L1 expression, indicating that activation of T cells can exert effects in prostate cancer patients. As immunotherapy has shown efficacy in a limited number of mCRPC patients, strategies to improve the efficacy of immunotherapy in this patient population are needed.

1.4.2 Abscopal effect and immunogenic cell death

The abscopal effect (*ab scopus* = away from target) can be observed during irradiation of tumor lesions and describes the immune-mediated response of metastatic lesions outside the radiation field (51). A similar immune response is seen after treatment with particular chemotherapeutic agents (52).

Although the abscopal effect is not fully understood, it is thought to start with the release of tumor-associated antigens (TAAs) from necrotic and apoptotic tumor cells upon tumor irradiation. Presentation of these TAAs to APCs leads to an activation of CD8+ T cells that subsequently attack both the primary tumor and metastatic lesions (53). In addition, irradiated tumor cells release cellular danger-associated molecular patterns (DAMPs) and cytokines that support immune cell traffic (54). The release of DAMPS and cytokines is the start of an event called immunogenic cell death (ICD) (55). There are three important DAMPs involved in ICD; endoplasmic reticulum chaperone calreticulin (CALR) is exposed on the surface of dying cells before apoptosis takes place, adenosine triphosphate (ATP) is secreted during the blebbing phase of apoptosis, and the nuclear protein high mobility group box 1 (HMGB1) is released upon plasma membrane permeabilization. These DAMPs promote the recruitment of immune cells, that subsequently secrete interferon-γ. Thereby, a change from a predominant tumor microenvironment cytokine profile towards an immunostimulatory profile is induced.

However, the abscopal effect and ICD remain a rare phenomenon, suggesting that the immunosuppressive capacity of the tumor(microenvironment) counteracts the radiation-induced activation of the immune system (54). By combining multiple treatment modalities, the treatment response might be augmented. Concurrent chemo-radiotherapy has been demonstrated to temporarily ablate immune-suppressive cells, induce the production of chemoattractants for effector T cells, and enhance antigen presentation towards such T cells (52, 56-58). Currently, various clinical trials investigating combination therapies with immune checkpoint inhibitors in mCRPC are under development (46).

1.4.3 Exploring the radium-223 induced immune response

<u>Blood based immune profiling</u>

In contrast to other tumors, mCRPC has in general a relatively low mutational load of 4.4 mutations/Mb(59). Only in a minority of mCRPC, higher mutation rates up to 50 mutations per Mb, which are likely due to defects in mismatch repair genes as MLH1 and MSH2, are found (59). In multiple cancers, a high mutational load predicts a better response to immunotherapy (60, 61). As radium-223, as a radiopharmaceutical, induces double stranded DNA breaks, it might make tumors more prone to immunotherapy. Currently, several studies are evaluating the efficacy of radium-223 in combination with immunotherapy in mCRPC patients.

Preliminary studies indicate that radium-223 induces immunological changes (62, 63). Exposure of prostate cancer cell lines to a sublethal dose of radium-223 enhances T-cell mediated lysis of tumor cells by antigen specific CD8+ cytotoxic T lymphocytes (CTLs) and induces phenotypic changes (62). Additionally, one radium-223 injection in patients with prostate cancer results in a decrease in the frequency of PD-1 expressing cytotoxic T cells (63). As understanding of treatment efficacy of radium-223 is essential for the rational development of combination therapies, further research into the radium-223-induced immune response is necessary. By tracking immune cells during treatment over time, we expect to increase our knowledge on the radium-223 induced immune response.

Imaging PD-L1 signaling with PET

As mentioned before, binding of PD-L1 to the PD-1 receptor on T cells inhibits activation of the immune system, thereby inhibiting the anti-tumor response. Radium-223 is thought to activate the immune system. Changes in PD-1/L1 signaling might reflect this activation.

Although PD-L1 expression can be determined on tumor tissue by immunohistochemistry, results are conflicting with regard to its predictive value during PD-1/PD-L1 blockade. This might be caused by differences in target (PD-1 or PD-L1) or methods, including differences in assays, definition of PD-L1 expression, used cutoffs or semi-quantitative analyses (46). As the value of immunohistochemistry appears rather limited, Bensch et al. have labeled a tracer dose of atezolizumab, a monoclonal antibody (mAb) targeting PD-L1, with zirconium-89 (^{89}Zr), which enables *in vivo* monitoring of PD-L1 expression using positron emission tomography (PET)(64). In patients with various malignancies (non-small cell lung cancer, bladder cancer and triple-negative breast cancer), PET using [^{89}Zr]atezolizumab showed heterogeneous uptake within and between patients, even in patients without PD-L1 expression as determined by immunohistochemistry. As whole body PET using [^{89}Zr]atezolizumab provides *in vivo* monitoring of PD-L1 expression at different metastatic sites, this technique may be useful to evaluate PD-L1 expression and local immune response during treatment.

1.5 Radium223Insight

In the current study (Radium223Insight), biomarkers for tumor and immune response during radium-223 are evaluated, thereby potentially contributing to a better understanding of immune responses during radium-223 and the rational design of future treatment strategies combining radium-233 with immunotherapy.

We hypothesize that:

1) Multidimensional biomarker collection enhances our understanding of tumor responses during radium-223, thereby potentially identifying biomarkers for early response.

2) Radium-223 induces DNA damage, resulting in immunogenic cell death and activation of the immune system, thereby making tumors more prone to immunotherapy.

2. OBJECTIVES

2.1 Primary objectives

1. To identify biomarkers for early identification of clinical benefit from radium-223 treatment in mCRPC patients.

2. To better understand immune response during radium-223 treatment in mCRPC patients.

2.2 Secondary objectives

1. To characterize the mutational and immunological profile of pre-treatment tumor genomes of mCRPC patients in relation to clinical benefit.

3. STUDY DESIGN

In this prospective, translational, multicenter study, mCRPC patients are treated with radium-223 according to standard of care. In order to identify early response biomarkers and improve our understanding of the radium-223 induced immune response, a variety of biomarkers will be assessed. The expected number of patients that will be recruited is 30.

3.1 Study treatment

Radium-223 is approved by the European Medicines Agency and Committee for the Assessment of Oncological Medicines (‘Commissie BOM’) for the treatment of mCRPC patients with symptomatic bone metastases without visceral metastases. All patients will receive six intravenous administrations of radium-223 according to standard of care at a dose of 55 kBq per kilogram of body weight, with an interval of four weeks during a period of 24 weeks.

3.2 Main study procedures

At baseline, during treatment with radium-223 and during follow-up several parameters will be obtained including clinical data, sequential blood samples (ctDNA, CTCs and immune markers), [^{68}Ga]PSMA PET-CT, [^{89}Zr]atezolizumab PET-CT, CT thorax/abdomen and bone scintigraphies. Tumor tissue will be obtained via a biopsy of a skeletal metastasis. This extensive collection of parameters provides a unique opportunity to map the characteristics of the mCRPC patients treated with radium-223. To minimize the patient’s burden, the study procedures will be minimized as much as possible by combining procedures and optimized scheduling of the visits.

3.2.1 Identification of response biomarkers

<u>Peripheral blood</u>

Circulating tumor cells (CTCs) will be isolated and enumerated with the CellSearch technology at baseline, after the third and sixth injection with radium-223 and upon progression. Moreover, we will obtain additional CTC samples for future molecular characterization.

As a novel response assessment tool we have set up a pipeline to use DNA Whole Genome Sequencing (WGS) as a detection method for structural variants. These structural variants can be detected and quantified in circulating tumor-derived DNA (ctDNA). Using serial circulating free DNA (cfDNA) sampling (from obtained plasma samples), tumor load can be tracked in real time.

<u>Imaging</u>

[^{68}Ga]PSMA PET-CT will be performed at baseline, after three and six injections of radium-223 and upon progression. Scans will be analyzed for change in SUV-value and number of lesions. A limited amount of blood will be drawn to determine the level of [^{68}Ga]-radioactivity in the blood. This is necessary for accurate imaging analysis. A PSMA PET-CT will be combined with a diagnostic CT, as a low-dose CT is not considered sufficient for response evaluation. [^{68}Ga]PSMA will be produced at the Department of Nuclear Medicine at Erasmus MC.

Additionally, a conventional bone scintigraphy will be performed as a reference standard at the same time points as the PSMA PET-CT to compare new and conventional imaging modalities. After the last administration of radium-223, bone scintigraphies and CTs will be performed as a part of regular follow-up to assess disease progression according to the PCWG3 criteria.

3.2.2 Immune profiling

<u>Peripheral blood</u>

Peripheral blood samples will be analyzed at baseline, during treatment and upon progression using multiparameter flow cytometry, with a focus on immune effector and suppressive cells, and the expression of T cell co-inhibitory molecules. Furthermore, serum samples will be assessed for levels of cytokines/chemoattractants. CITRUS (cluster identification, characterization, and regression), an automated, unsupervised bioinformatic approach will be used for the identification of stratifying subpopulations. Peripheral blood mononuclear cells (PBMC) will be stored for prospective analysis of T cell effector functions *in vitro* and T cell receptor (TCR) repertoire analysis.

<u>Imaging</u>

For in vivo monitoring of PD-L1 expression, sequential [^{89}Zr]atezolizumab PET-CT will be performed at baseline and after three injections of radium-223. Scans will be analyzed for change in SUV-value. A limited amount of blood will be drawn to determine the level of [^{89}Zr]-radioactivity in the blood. This is necessary for accurate imaging analysis.

[^{89}Zr]atezolizumab will be produced in collaboration with the Departments of Medical Oncology and Nuclear Medicine of UMCG.

<u>Tumor tissue</u>

Tumor tissue from a skeletal metastasis will be obtained via a bone biopsy. Only patients with a safely accessible lesion will be included for a histological biopsy and biopsies will be

performed according to local safety guidelines. Location of the biopsy will be based on the PSMA PET-CT. The biopsy procedure itself will be CT-guided. Formaline-fixed paraffin-embedded (FFPE) and fresh frozen tissue will be collected. A part of the patients will be asked for an optional on-treatment or post-treatment biopsy.

FFPE tissues will be analyzed by *in situ* multiplex immunofluorescence using automated quantitative pathology imaging (Vectra system, PerkinElmers), thereby identifying tumor epithelial and stromal cells, effector immune cells, T cell co-inhibition, T cell recruitment and immune-suppressive cells. The Vectra system enables these analyses in tissue without further manipulation.

3.2.3 Molecular profiling

In addition to FFPE tissue for immunofluorescence, fresh frozen tissue from a skeletal metastasis and a blood sample will be obtained for DNA isolation of both tumor and normal white blood cells. DNA will be submitted for whole genome sequencing and data will be analyzed for immune and molecular profiling. The genome data will be used to explore potential correlations between specific pathway aberrations, total number of somatic variants and pattern of mutational load characteristic of the DNA mutational agent and clinical outcome. Moreover, we will perform whole genome RNA sequencing, an essential part of the genomic analysis to identify tumor specific neoantigens.

4. STUDY POPULATION

4.1 Population (base)

mCRPC patients with symptomatic bone metastases, but without visceral metastases, considered eligible for radium-223 treatment. Within eighteen months, we expect to enroll 30 patients.

4.2 Inclusion criteria

- Age ≥ 18 years
- Histologically confirmed, progressive prostate cancer during ADT. Castration-resistant disease is defined as a serum testosterone level of 50 ng per deciliter or lower (≤1.7 nmol per liter) after bilateral orchiectomy or during maintenance treatment consisting of androgen-ablation therapy with a luteinizing hormone–releasing hormone agonist.
- Baseline PSA level of ≥ 2 ng per milliliter with evidence of progressively increasing PSA values (two consecutive increases over the previous reference value)
- Prior treatment with at least two other approved agents for metastatic prostate cancer, unless the patient is not able and/or willing to receive other treatments.
- Eastern Cooperative Oncology Group (ECOG) performance-status score of 0 or 1 (on a scale of 0 to 5, with 0 indicating no symptoms and full activity and higher scores indicating greater functional compromise)
- Life expectancy of 6 months or longer
- Adequate hematologic, renal, and liver function, including trombocytes >100x10^9/L, granulocytes >1.5x10^9/L, Hb >6.2 mmol/L.

4.3 Exclusion criteria

- Prior chemotherapy, other than docetaxel.
- Previous hemibody external radiotherapy or systemic radiotherapy with radioisotopes within the previous 24 weeks.
- A blood transfusion or use of erythropoietin-stimulating agents within the previous 4 weeks
- Pathological lymphadenopathy > 1.5 cm in the short-axis diameter on CT or MRI. [^{68}Ga]-PSMA positive lymph nodes at baseline are allowed.
- A history of presence of visceral metastases on CT or MRI. Patients with [^{68}Ga]-PSMA positive lesions that are highly suspected for visceral metastases and are retrospectively visible on CT or MRI should be excluded as well.
- Imminent or established spinal cord compression on CT or MRI.
- A second active malignancy.

4.4 Sample size calculation

As this is an exploratory hypothesis-generating study, no formal power calculation is possible. Due to financial restrictions the sample size has been set at 30 patients. We expect this sample size to be enough for answering the exploratory research questions of this study.

5. TREATMENT OF SUBJECTS

5.1 Investigational product

In this study, mCRPC patients will be treated with radium-223 according to standard of care. No investigational medicinal products will be used in this study.

5.2 Use of co-intervention

Not applicable

5.3 Escape medication

Not applicable

6. INVESTIGATIONAL PRODUCT

Not applicable.

7. NON-INVESTIGATIONAL PRODUCT

7.1 Name and description of non-investigational product(s)

Radium-223

Radium-223 (Xofigo) is an alpha-emitting radiopharmaceutical, that mimics calcium by binding to hydroxyapatite in the bones. Patients will be treated with radium-223 according to standard of care.

$[^{68}Ga]$PSMA

$[^{68}Ga]$PSMA will be used as a diagnostic tracer for PET-CT to image prostate cancer cells. $[^{68}Ga]$PSMA PET CT is already used in clinical practice and will be performed according to local guidelines (65).

$[^{89}Zr]$atezolizumab

$[^{89}Zr]$atezolizumab will be used as a diagnostic tracer for PET-CT to image PD-L1 signaling. Recently the results of the first in-human $[^{89}Zr]$atezolizumab PET CT imaging were published by Bensch et al(64). $[^{89}Zr]$atezolizumab PET CT imaging appeared to be safe with only one tracer related low-grade adverse event.

7.2 Dosages, dosage modifications and method of administration

Radium-223

The registered dose of radium-223 is 55 kBq per kilogram of body weight intravenously per injection. Six injections are given with an interval of four weeks.

$[^{68}Ga]$PSMA

Patients will receive $[^{68}Ga]$PSMA intravenously prior to the PSMA PET-CT. The dosage of $[^{68}Ga]$PSMA will be 1.5 MBq/kg.

$[^{89}Zr]$atezolizumab

Patients will receive 37 MBq ^{89}Zr-atezolizumab (~11 mg, <1% of therapeutic dose), which will be injected intravenously prior to the $[^{89}Zr]$atezolizumab PET-CT.

7.3 Preparation and labelling of Non Investigational Medicinal Product

Radium-223

Radium-223, as a standard of care treatment, will be prepared according to the relevant GMP guidelines.

$[^{68}Ga]$PSMA

$[^{68}Ga]$PSMA will be prepared according to the relevant GMP guidelines. Specific instructions for preparation can be found in local guidelines(65).

[^{89}Zr]atezolizumab

[^{89}Zr]atezolizumab will be prepared according to the relevant GMP guidelines. Additional information can be found in the corresponding IMPD (66).

8. METHODS

8.1 Study parameters/endpoints

8.1.1 Main study endpoints

Exploratory analysis of multiple biomarkers in relation to failure-free survival, defined as time to next line of treatment, best supportive care or death. Next line of treatment or best supportive care will be started upon clinical, biochemical and/or radiological signs of progression according to the PCWG3 criteria.

Biomarkers:

a) Blood based biomarkers:
   a. Conventional markers

      tALP decline from baseline to week 12 (confirmed > 3 weeks from week 12) correlates with an increased failure-free survival, and other endpoints.

   b. CTC's

      <5 CTC's/7.5 ml at baseline correlates with an increased failure-free survival, and other endpoints.

   c. ctDNA

      >50% decline in the allelic fraction of mutant tumor-derived DNA within total cell-free DNA during treatment correlates with an increased failure-free survival, and other endpoints.

   d. Immune profiling data

b) Imaging based biomarkers:
   a. Bone scintigraphy

      2+2 rule correlates with a decreased failure-free survival, and other endpoints.

   a. [^{68}Ga]PSMA PET-CT

      Reduction in SUV_{max} of >30% in the hottest baseline lesion correlates with an increased failure-free survival, and other endpoints.

   b. [^{89}Zr]atezolizumab PET-CT

2. Exploratory analysis of the immune response during radium-223 treatment in relation to failure-free survival as defined above:

   a) Blood based analysis of immune cells and markers

Calculation of absolute numbers of immune cells by multi-color flowcytometry, and other endpoints.

b) In vivo monitoring of PD-L1 signaling by [^{89}Zr]atezolizumab PET-CT

Analysis of SUV_{max} on [^{89}Zr]atezolizumab PET-CT over time, and other endpoints.

c) In situ multiplex immune fluorescence of FFPE using automated quantitative pathology imaging.

Quantification of PD-L1 positive tumor cells and immune cells in biopsy tissue, and other endpoints.

8.1.2 Secondary study endpoints

1. Characterization of the mutational and immunological profile of pre-treatment tumor genome in relation to failure free survival as defined above.

2. Overall survival

3. Skeletal related events

8.2 Randomisation, blinding and treatment allocation

Randomisation is not applicable, as this is an open-label, single-arm trial. Treating physicians will be blinded for the results of CTC's, ctDNA and immunology analysis, CT and bone scintigraphy in week 11, PSMA PET-CT in week 11 and 24 and [^{89}Zr]atezolizumab-PET-CT at baseline and in week 11, as these biomarkers are not considered standard of care in this setting and may affect treatment planning inadequately. The PSMA PET-CT scans at baseline and at progression will not be blinded. The baseline scan will be used to guide the bone biopsy. Additionally, if the baseline PSMA PET-CT detects visceral lesions that are highly suspected for metastases and can be retrospectively visualized on CT or MRI, patients should be excluded from the study. Lymphadenopathy on PSMA PET-CT is accepted. The PSMA PET-CT at progression won't be blinded as well as this scan will be performed after progression is identified and thus won't influence the primary endpoint. Besides, this scan can be of additional value for future treatment planning. Development of visceral metastases on CT will be communicated with the treating physician as well.

8.3 Study procedures

Study procedures are summarized in Figure 1.

	Baseline	During treatment Radium-223 at week 0, 4, 8, 12, 16 and 20					End of treatment	Follow-up Repeat until progression*				Upon progression
**WEEKS**	**-1**	**3**	**7**	**11**	**15**	**19**	**24**	**30**	**36**	**42**	**48**	
**BLOOD**												
Conventional	●	●	●	●	●	●	●	●	●	●	●	●
CTC	●			●			●					●
ctDNA	●	●	●	●	●	●	●		●		●	●
Immunology	●	●		●			●					●
Biopsy related	●			○			○					
**IMAGING**												
Diagnostic CT-thorax/abdomen	●			●			●		●		●	●
Bone scintigraphy	●			●			●		●		●	●
[68Ga]PSMA-PET-CT	●			●			●					●
[89Zr]atezolizumab-PET-CT	●			●								
**TISSUE**												
PSMA-guided bone biopsy	●			○			○					

● Standard
○ Optional

*function, including LDH, ALP, PSA, testosterone, APPT and PT-INR. Conventional blood at remaining time points consists of total blood count, PSA, ALP and LDH. Required volumes and tubes for study blood draws: 1x 10 ml EDTA + 1x10 ml CellSave (CTC), 2x10ml CellSave (ctDNA), 2x10 ml EDTA (immunology) and 1x10ml CellSave (biopsy related). Blood draws take place 1-2 weeks before each radium-223 injection. Diagnostic CT-thorax/abdomen and bone scintigraphy at baseline, end of treatment and during follow-up are standard of care. The pre-treatment biopsy is standard, the on-treatment and post-treatment biopsies are optional. FFPE and fresh frozen tissue will be collected. *During follow-up, blood will be checked every six weeks and imaging will be performed every 12 weeks according to standard of care. After 48 weeks blood will be checked every 12 weeks as well. This scheme continues until progression of disease takes place.*

8.3.1 Baseline screening

- Written informed consent
- Age
- Medical history, including tumor characteristics (Gleason score, clinical and pathological state, etc), PSA and testosterone levels, prior imaging, previous prostate cancer therapies (type, dose, date) and supportive care for cancer-related bone pain (radiotherapy or use of analgesic medication) or anemia (blood transfusion or use of erythropoietin-stimulating agents).
- Medication review
- ECOG performance status
- Laboratory examinations: hematologic, liver and renal function, testosterone, PSA (performed in the last 28 days).
- Diagnostic CT-thorax/abdomen to exclude the presence of visceral metastases (performed in the last 42 days).
- Bone scintigraphy to confirm four or more bone metastases (performed in the last 42 days)

8.3.2 At baseline, prior to administration of radium-223

- Laboratory examinations (1-2 weeks before start treatment)
    - Conventional: Hb, thrombocytes, leukocytes, PSA, LDH, ALP, APPT, PT-INR (only if laboratory examination at baseline is older than 2 weeks before start treatment) (1x EDTA, 1x lithium-heparin, 1x citrate)
    - Immunology: 2x 10 ml EDTA
    - CTC: 1x10 ml EDTA, 1x10 ml CellSave
    - ctDNA: 2x10 ml CellSave
    - Sequencing normal white blood cells (biopsy related): 1x10 ml BCT
- Conventional bone scintigraphy (if not performed in the last 42 days before start treatment)
- Conventional CT thorax/abdomen (if not performed in the last 42 days before start treatment)
- [^{68}Ga]PSMA-PET-CT
- [^{89}Zr]atezolizumab-PET-CT
- PSMA-PET-CT guided biopsy of skeletal metastasis

8.3.3 Before the second cycle of radium-223

- Laboratory examinations (1-2 weeks before second injection)

  - Conventional: Hb, thrombocytes, leukocytes, PSA, LDH, ALP (1x EDTA, 1x lithium-heparin)
  - Immunology: 2x 10 ml EDTA
  - ctDNA: 2x10 ml CellSave

8.3.4 Before the third cycle of radium-223

- Laboratory examinations (1-2 weeks before third injection)
  - Conventional: Hb, thrombocytes, leukocytes, PSA, LDH, ALP (1x EDTA, 1x lithium-heparin)
  - ctDNA: 2x10 ml CellSave

8.3.5 Before the fourth cycle of radium-223

- Laboratory examinations (1-2 weeks before fourth injection)
  - Conventional: Hb, thrombocytes, leukocytes, PSA, LDH, ALP (1x EDTA, 1x lithium-heparin)
  - Immunology: 2x 10 ml EDTA
  - CTC: 1x10 ml EDTA, 1x10 ml CellSave
  - ctDNA: 2x10 ml CellSave
  - *Optional: sequencing normal white blood cells (biopsy related): 1x10 ml BCT*
- Conventional bone scintigraphy
- Conventional CT thorax/abdomen
- [^{68}Ga]PSMA-PET-CT
- [^{89}Zr]atezolizumab-PET-CT
- *Optional: PSMA-PET-CT guided biopsy of skeletal metastasis*

8.3.6 Before the fifth and sixth cycle of radium-223

- Laboratory examinations (1-2 weeks before next injection)
  - Conventional: Hb, thrombocytes, leukocytes, PSA, LDH, ALP (1x EDTA, 1x lithium-heparin)
  - ctDNA: 2x10 ml CellSave

8.3.7 Four weeks after the last radium-223 injection

- Laboratory examinations
  - Conventional: Hb, thrombocytes, leukocytes, PSA, LDH, ALP (1x EDTA, 1x lithium-heparin)
  - Immunology: 2x 10 ml EDTA
  - CTC: 1x10 ml EDTA, 1x10 ml CellSave
  - ctDNA: 2x10 ml CellSave

    - *Optional: sequencing normal white blood cells (biopsy related): 1x10 ml BCT*
- Conventional bone scintigraphy
- Conventional CT thorax/abdomen
- [^{68}Ga]PSMA-PET-CT
- *Optional: PSMA-PET-CT guided biopsy of skeletal metastasis*

8.3.8 Follow-up

After completion of radium-223 treatment, follow-up will be performed according to standard of care. Blood will be checked every six weeks for biochemical progression. After 48 weeks without progression, blood will be checked every 12 weeks. Blood samples for ctDNA will be obtained every 12 weeks. Bone scintigraphies and diagnostic CTs will be performed every three months until progression of disease occurs.

The following parameters will be recorded:

- Failure-free survival, defined as time to next line of treatment, best supportive care or death.
- Overall survival
- Skeletal related events

8.3.9 Progression of disease

Upon failure defined as start of next line of treatment or best supportive care due to clinical, biochemical and/or radiological progression according to the PCWG3 criteria, the following biomarkers will be obtained:

- Laboratory examinations
    - Conventional: Hb, thrombocytes, leukocytes, PSA, LDH, ALP (1x EDTA, 1x lithium-heparin)
    - Immunology: 2x 10 ml EDTA
    - CTC: 1x10 ml EDTA, 1x10 ml CellSave
    - ctDNA: 2x10 ml CellSave
- Conventional bone scintigraphy
- Conventional CT thorax/abdomen
- [^{68}Ga]PSMA-PET-CT

8.4 Withdrawal of individual subjects

Subjects can leave the study at any time for any reason if they wish to do so without any consequences. The investigator can decide to withdraw a subject from the study for urgent medical reasons.

8.4.1 Specific criteria for withdrawal

Not applicable.

8.5 Replacement of individual subjects after withdrawal

Patients who have withdrawn from the study will be replaced if radium-223 treatment has not been administered yet.

8.6 Follow-up of subjects withdrawn from treatment

When patients are withdrawn from treatment (after the first dose of radium-223) because of medical reasons, like toxicity, study procedures, e.g. blood draws and imaging, will be continued until next line of treatment as long as this does not intervene with the patient's well-being.

8.7 Premature termination of the study

As patients are treated according to standard-of-care, no premature termination of the study is foreseen.

9. SAFETY REPORTING

9.1 Temporary halt for reasons of subject safety

In accordance to section 10, subsection 4, of the WMO, the sponsor will suspend the study if there is sufficient ground that continuation of the study will jeopardise subject health or safety. The sponsor will notify the accredited METC without undue delay of a temporary halt including the reason for such an action. The study will be suspended pending a further positive decision by the accredited METC. The investigator will take care that all subjects are kept informed.

9.2 AEs, SAEs and SUSARs

9.2.1 Adverse events (AEs)

Adverse events are defined as any undesirable experience occurring to a subject during the study, whether or not considered related to radium-223. All adverse events > grade 3, occurring from the day of randomization until 30 days after the last radium cycle, reported spontaneously by the subject or observed by the investigator or his staff will be recorded.

9.2.2 Serious adverse events (SAEs)

A serious adverse event is any untoward medical occurrence or effect that

- results in death;
- is life threatening (at the time of the event);
- requires hospitalisation or prolongation of existing inpatients' hospitalisation;
- results in persistent or significant disability or incapacity;
- is a congenital anomaly or birth defect; or
- any other important medical event that did not result in any of the outcomes listed above due to medical or surgical intervention but could have been based upon appropriate judgement by the investigator.

An elective hospital admission, hospital admission due to progressive disease and side effects related to radium-223 treatment will not be considered as a serious adverse event.

All serious adverse events, occurring from the day of randomization until 30 days after the last radium cycle, reported spontaneously by the subject or observed by the investigator or his staff will be recorded. The local investigator will report the SAEs to the coordinating investigator within 48 hours after becoming aware of the SAE. The coordinating investigator will report the SAEs through the web portal *ToetsingOnline* to the accredited METC that approved the protocol, within 7 days of first knowledge for SAEs that result in death or are life threatening followed by a period of maximum of 8 days to complete the initial preliminary report. All other

SAEs will be reported within a period of maximum 15 days after the sponsor has first knowledge of the serious adverse events.

9.2.3 Suspected unexpected serious adverse reactions (SUSARs)

Adverse reactions are all untoward and unintended responses to an investigational product related to any dose administered.

Unexpected adverse reactions are SUSARs if the following three conditions are met:

1. the event must be serious (see chapter 9.2.2);
2. there must be a certain degree of probability that the event is a harmful and an undesirable reaction to the medicinal product under investigation, regardless of the administered dose;
3. the adverse reaction must be unexpected, that is to say, the nature and severity of the adverse reaction are not in agreement with the product information as recorded in:

- Summary of Product Characteristics (SPC) for an authorised medicinal product;
- Investigator's Brochure for an unauthorised medicinal product.

The sponsor will report expedited the following SUSARs through the web portal *ToetsingOnline* to the METC

- SUSARs that have arisen in the clinical trial that was assessed by the METC;
- SUSARs that have arisen in other clinical trials of the same sponsor and with the same medicinal product, and that could have consequences for the safety of the subjects involved in the clinical trial that was assessed by the METC.

The remaining SUSARs are recorded in an overview list (line-listing) that will be submitted once every half year to the METC. This line-listing provides an overview of all SUSARs from the study medicine, accompanied by a brief report highlighting the main points of concern.

The expedited reporting of SUSARs through the web portal ToetsingOnline is sufficient as notification to the competent authority.

The expedited reporting will occur not later than 15 days after the sponsor has first knowledge of the adverse reactions. For fatal or life threatening cases the term will be maximal 7 days for a preliminary report with another 8 days for completion of the report.

9.3 Annual safety report

In addition to the expedited reporting of SUSARs, the sponsor will submit, once a year throughout the clinical trial, a safety report to the accredited METC and competent authority.

This safety report consists of:

- a list of all suspected (unexpected or expected) serious adverse reactions, along with an aggregated summary table of all reported serious adverse reactions, ordered by organ system, per study;
- a report concerning the safety of the subjects, consisting of a complete safety analysis and an evaluation of the balance between the efficacy and the harmfulness of the medicine under investigation.

9.4 Follow-up of adverse events

All AEs will be followed until they have abated, or until a stable situation has been reached. Depending on the event, follow up may require additional tests or medical procedures as indicated, and/or referral to the general physician or a medical specialist.

9.5 Data Safety Monitoring Board (DSMB) / Safety Committee

There will be no Data Safety Monitoring Board for this study.

10. STATISTICAL ANALYSIS

The primary objectives of this study are the exploration of multiple biomarkers for the early response evaluation of radium-223 treatment and the improvement of our understanding of the radium-223 induced immune response in mCRPC patients.

10.1 Primary study parameter(s)

Biomarker and immune parameters will be presented mainly as quantitative data. Absolute numbers at baseline and during treatment will be described, as well as relative changes in parameters over time. Furthermore, biomarkers measured at baseline will be related to failure free survival by means of Cox regression analysis. For repeatedly measured biomarkers time-dependent Cox regression will be used.

10.2 Secondary study parameter(s)

The characterization of the mutational and immunological profile of pre-treatment tumor genome will be related to failure free survival with Cox regression analysis.
For overall survival and skeletal related events similar analyses will be performed as for failure free survival.

11. ETHICAL CONSIDERATIONS

11.1 Regulation statement

The investigator will ensure that this study is conducted to the standards of Good Clinical Practice, in full conformance with the "Declaration of Helsinki" (latest amendment), the Dutch laws and regulations and with the W.M.O. ("Wet Medisch-wetenschappelijk Onderzoek met mensen") in particular.

11.2 Recruitment and consent

Patients will be asked to participate in the study when they are eligible for radium treatment. Patients will be informed on the aim, the procedures, the risks and benefits of the study by the treating physician or study coordinator. Besides, they will receive written information (patient information form). After the study information is given, patients will receive enough time to consider whether they want to join the study or not. Before study procedures will start, a written informed consent will be signed by the patient and responsible physician/study coordinator. The patient will be provided with a copy of the signed written informed consent. The patient is allowed to withdraw from the study at any time without prejudicing future medical treatment.

11.3 Objection by minors or incapacitated subjects

Not applicable.

11.4 Compensation for injury

The sponsor/investigator has a liability insurance which is in accordance with article 7 of the WMO. The sponsor (also) has an insurance which is in accordance with the legal requirements in the Netherlands (Article 7 WMO). This insurance provides cover for damage to research subjects through injury or death caused by the study;

I. € 650.000,- (i.e. six hundred and fifty thousand Euro) for death or injury for each subject who participates in the research;

II. € 5.000.000,- (i.e. five million Euro) for death or injury for all subjects who participate in the research;

III. € 7.500.000,- (i.e. seven million five hundred thousand Euro) for the total damage incurred by the organization for all damage disclosed by scientific research for the Sponsor as 'verrichter' in the meaning of said Act in each year of insurance coverage.

The insurance applies to the damage that becomes apparent during the study or within 4 years after the end of the study.

11.5 Incentives

Patients can receive a reimbursement for parking and travel costs on days they travel to the hospital for study procedures only.

12. ADMINISTRATIVE ASPECTS, MONITORING AND PUBLICATION

12.1 Handling and storage of data and documents

After the written informed consent has been signed, the local investigator will fill in the registration form and send this to the clinical trial center of the department of Medical Oncology. The clinical trial center will create a unique non-identifiable number for each patient. The key to the subject number will be safeguarded by the local investigator. The registration form will at least include the patient’s subject number, the name of the treating hospital and investigator, a statement that the patient meets all selection criteria and the date of the written informed consent. All patient samples will be coded with the patient’s subject number.

The original signed informed consent will be stored on site in the Investigators Site File. Patient data will be collected in an electronic CRF in a digital database and will be available online to complete on site by the study coordinator. Collected data, essential documents, source documents and human materials will be stored for at least 15 years in such a manner that they are protected from accidental loss and can be easily retrieved for review. Human materials which remain after completion of the study will be kept completely anonymized. Essential documents are those documents that permit evaluation of the conduct of a trial and the quality of the data produced. The essential documents may be subject to, and should be available for audit by the Sponsor’s auditor and inspection by the regulatory authorities.

12.2 Monitoring and Quality Assurance

The risk of adverse events related to radium-223 treatment is low. Following the guidelines by the NFU (Dutch Federation of University Medical Centers) regarding quality insurance in human research (“Kwaliteitsborging van mensgebonden onderzoek”), the risks of this study can be considered negligible (small chance of minor damage). According to this negligible risk a ‘minimal monitoring’ will be performed as described by the NFU by the Clinical Trial Center of the Erasmus MC. Monitoring will be performed according to the study specific Monitoring plan.

12.3 Amendments

A ‘substantial amendment’ is defined as an amendment to the terms of the METC application, or to the protocol or any other supporting documentation, that is likely to affect to a significant degree:

- the safety or physical or mental integrity of the subjects of the trial;
- the scientific value of the trial;
- the conduct or management of the trial; or

- the quality or safety of any intervention used in the trial.

All substantial amendments will be notified to the METC and to the competent authority.
Non-substantial amendments will not be notified to the accredited METC and the competent authority, but will be recorded and filed by the sponsor.

12.4 Annual progress report

The sponsor/investigator will submit a summary of the progress of the trial to the accredited METC once a year. Information will be provided on the date of inclusion of the first subject, numbers of subjects included and numbers of subjects that have completed the trial, serious adverse events/ serious adverse reactions, other problems, and amendments.

12.5 Temporary halt and (prematurely) end of study report

The sponsor will notify the accredited METC and the competent authority of the end of the study within a period of 90 days. The end of the study is defined as the last patient’s last visit.

The sponsor will notify the METC immediately of a temporary halt of the study, including the reason of such an action.

In case the study is ended prematurely, the sponsor will notify the accredited METC and the competent authority within 15 days, including the reasons for the premature termination.

Within one year after the end of the study, the investigator/sponsor will submit a final study report with the results of the study, including any publications/abstracts of the study, to the accredited METC and the Competent Authority.

12.6 Public disclosure and publication policy

The sponsor of this investigator driven study is the Erasmus Medical Centre Rotterdam. The study is financially sponsored by Bayer HealthCare.

Independent of the study’s outcome, results will be submitted for publication in a peer reviewed scientific journal, unless the trial was terminated prematurely and did not yield sufficient data for a publication. The final results of this study will be analyzed by the coordinating investigator under supervision of the principal investigator. Before submission to a scientific journal, a draft manuscript will be send to all co-authors for approval.

13. STRUCTURED RISK ANALYSIS

13.1 Potential issues of concern

Potential issues of concern are addressed in section 13.2. As radium-223 is a standard treatment for mCRPC patients with bone metastases, this chapter is not applicable.

13.2 Synthesis

Treatment with radium-223 improves OS in mCRPC patients with bone metastases. However, traditional parameters fail to measure this response. Since response evaluation is expected to critically contribute to both understanding treatment efficacy and guiding treatment decision making, the current study aims to improve response evaluation by multidimensional biomarker research. In addition, we aim to improve the knowledge on the effect of radium-223 on the immune system in order to enable the rational development of combination strategies including radium-223.

In this study, patients receive radium-223 according to standard of care. During treatment multiple biomarkers and immune parameters are collected. Participation in this study requires additional blood draws, imaging, and a pre-treatment bone biopsy.

Total needed blood volume varies between 30-80 ml per blood draw (including standard laboratory examinations). Blood draws will take place before each cycle (every four weeks), during follow-up and upon progression in combination with regular blood draws. Additionally, a limited amount of blood will be drawn to measure the radioactivity of the tracers in blood, which is necessary for good imaging analysis. The risk of this limited amount of blood being drawn is minimal.

Additionally, patients will undergo four imaging modalities during treatment: three bone scintigraphies, three CT scans of the thorax and abdomen, CT-guidance during the bone biopsy, three [^{68}Ga]PSMA PET-CT scans and two [^{89}Zr]atezolizumab PET-CT scans. The bone scintigraphy and the CT-thorax/abdomen at baseline and at the end of treatment are standard of care. The remaining scans will give an additional radiation burden of 80 mSv, divided over 24 weeks. During follow-up, CT scans and bone scintigraphies will be performed according to standard of care. At disease progression, an additional [^{68}Ga]PSMA PET-CT will be performed, which accounts for 6 mSv. Based on the ALSYMPCA trial, the patient population in this study will have a median age of 71 and a median survival of approximately 15 months. Although the absolute additional radiation burden is considerable, this is only about 3% of the estimated effective dose of the radium-223 treatment, which accounts for approximately 3000 mSv. Considering the age of the patients, the prognosis, the relative small additional radiation

burden on top of the standard radium-223 treatment and the substantial benefit of this study for future treatment of prostate cancer patients, the radiation burden of the imaging protocol is considered justified.

The risk of the bone biopsy is considered low (1). All safety measures and procedures will be performed according to local guidelines.

14. REFERENCES

1. S. B, G.A. C, Gadellaa-Van Hooijdonk CG, Weeber F, Numan IJ, Bruggink AH, et al. Implementation of aMulticenter Biobanking Collaboration for Next Generation Sequencing-Based Biomarker Discovery Based on Fresh Frozen Pretreatment Tumor Tissue Biopsies. The Oncologist. 2017;22:33-40.
2. Nederland IK. Cijfers over kanker. 2017.
3. Ward JF, Moul JW. Rising prostate-specific antigen after primary prostate cancer therapy. Nat Clin Pract Urol. 2005 Apr;2(4):174-82.
4. Nederland IK. Deelrapportage voor prostaatkanker. 2014.
5. Ibrahim T, Flamini E, Mercatali L, Sacanna E, Serra P, Amadori D. Pathogenesis of osteoblastic bone metastases from prostate cancer. Cancer. 2010 Mar 15;116(6):1406-18.
6. Sottnik JL, Keller ET. Understanding and targeting osteoclastic activity in prostate cancer bone metastases. Curr Mol Med. 2013 2013 May 1;13(4):626-39.
7. Howard LE, De Hoedt AM, Aronson WJ, Kane CJ, Amling CL, Cooperberg MR, et al. Do skeletal-related events predict overall survival in men with metastatic castration-resistant prostate cancer? Prostate Cancer Prostatic Dis. 2016 Dec;19(4):380-4.
8. James ND, Sydes MR, Clarke NW, Mason MD, Dearnaley DP, Spears MR, et al. Addition of docetaxel, zoledronic acid, or both to fi rst-line long-term hormone therapy in prostate cancer (STAMPEDE): survival results from an adaptive, multiarm, multistage, platform randomised controlled trial. Lancet. 2016;387:1163-77.
9. Lavaud P, Gravis G, Foulon S, Joly F, Oudard S, Priou F, et al. Anticancer Activity and Tolerance of Treatments Received Beyond Progression in Men Treated Upfront with Androgen Deprivation Therapy With or Without Docetaxel for Metastatic Castration-naive Prostate Cancer in the GETUG-AFU 15 Phase 3 Trial. Eur Urol. 2017 Oct 23.
10. Sweeney CJ, Chen YH, Carducci M, Liu G, Jarrard DF, Eisenberger M, et al. Chemohormonal Therapy in Metastatic Hormone-Sensitive Prostate Cancer. N Engl J Med. 2015 Aug 20;373(8):737-46.
11. Sharifi N, Gulley JL, Dahut WL. Androgen Deprivation Therapy for Prostate Cancer. JAMA. 2005 July 13, 2005;294(2):238-44.
12. Tannock IF, De Wit R, Berry WR, Horti J, Pluzanska A, Chi KN, et al. Docetaxel plus Prednisone or Mitoxantrone plus Prednisone for Advanced Prostate Cancer. The New England Journal of Medicine. 2004;351:1502-12.
13. Fizazi K, Scher HI, Molina A, Logothetis CJ, Chi KN, Jones RJ, et al. Abiraterone acetate for treatment of metastatic castration-resistant prostate cancer: fi nal overall survival analysis of the COU-AA-301 randomised, double-blind, placebo-controlled phase 3 study. Lancet Oncol. 2012;13:983-92.
14. Beer TM, Armstrong AJ, Rathkopf DE, Loriot Y, Sternberg CN, Higano CS, et al. Enzalutamide in metastatic prostate cancer before chemotherapy. N Engl J Med. 2014 Jul 31;371(5):424-33.
15. Parker C, Nilsson S, Heinrich D, Helle SI, O'Sullivan JM, Fossa SD, et al. Alpha emitter radium-223 and survival in metastatic prostate cancer. N Engl J Med. 2013 Jul 18;369(3):213-23.
16. Scher HI, Fizazi K, Saad F, Taplin ME, Sternberg CN, Miller K, et al. Increased survival with enzalutamide in prostate cancer after chemotherapy. N Engl J Med. 2012 Sep 27;367(13):1187-97.
17. de Bono JS, Oudard S, Ozguroglu M, Hansen S, Machiels J, Kocak I, et al. Prednisone plus cabazitaxel or mitoxantrone for metastatic castration-resistant prostate cancer progressing after docetaxel treatment: a randomised open-label trial. Lancet 2010;376:1147-54.
18. Salazar OM, Sandhu T, Da Motta NW, Angeles Perez Escutia M, Lanzos-Gonzales E, Mouelle-Sone A, et al. Fractionated half-body irradiation (HBI) for the rapid palliation of widespread, symptomatic, metastatic bone disease: a randomized pahse III trial of the international atomic energy agency (IAEA). Int J Radiation Oncology Biol Phys 2001;50:765-75.

19.Jong JM, Oprea-Lager DE, Hooft L, de Klerk JM, Bloemendal HJ, Verheul HM, et al. Radiopharmaceuticals for Palliation of Bone Pain in Patients with Castration-resistant Prostate Cancer Metastatic to Bone: A Systematic Review. Eur Urol. 2016 Sep;70(3):416-26.
20.Bruland OS, Nilsson S, Fisher DR, Larsen RH. High-linear energy transfer irradiation targeted to skeletal metastases by the alpha-emitter 223Ra: adjuvant or alternative to conventional modalities? Clin Cancer Res. 2006 Oct 15;12(20 Pt 2):6250s-7s.
21.Riegman PHJ, Vlietstra RJ, Van der Korput JAGM, Brinkmann AO, Trapman J. The promotor of the prostate-specific antigen gene contains a functional androgen responsive element. Molecular Endocrinology. 1991;5(12):1921-30.
22.Halabi S, Armstrong AJ, Sartor O, de Bono J, Kaplan E, Lin CY, et al. Prostate-specific antigen changes as surrogate for overall survival in men with metastatic castration-resistant prostate cancer treated with second-line chemotherapy. J Clin Oncol. 2013 Nov 1;31(31):3944-50.
23.Sartor O, Coleman RE, Nilsson S, Heinrich D, Helle SI, O'Sullivan JM, et al. An exploratory analysis of alkaline phosphatase, lactate dehydrogenase, and prostate-specific antigen dynamics in the phase 3 ALSYMPCA trial with radium-223. Ann Oncol. 2017 May 1;28(5):1090-7.
24.deSouza NM, Liu Y, Chiti A, Oprea-Lager D, Gebhart G, Van Beers BE, et al. Strategies and technical challenges for imaging oligometastatic disease: Recommendations from the European Organisation for Research and Treatment of Cancer imaging group. Eur J Cancer. 2018 Mar;91:153-63.
25.Azad GK, Taylor B, Rubello D, Colletti PM, Goh V, Cook GJ. Molecular and Functional Imaging of Bone Metastases in Breast and Prostate Cancers: An Overview. Clin Nucl Med. 2016 Jan;41(1):e44-50.
26.Scher HI, Morris MJ, Stadler WM, Higano C, Basch E, Fizazi K, et al. Trial Design and Objectives for Castration-Resistant Prostate Cancer: Updated Recommendations From the Prostate Cancer Clinical Trials Working Group 3. J Clin Oncol. 2016 Apr 20;34(12):1402-18.
27.Silver DA, Pellicer I, Fair WR, Heston WDW, Cordon-Cardo C. Prostate-specific membrane antigen expression in normal and malignant human tissues. Clinical Cancer Research. 1997;3:81-5.
28.Wright GL, Haley C, Becket ML, Schellhammer PF. Expression of prostate-specific membrane antigen in normal, benign, and malignant prostate tissues. Urol Oncol. 1995;1:18-28.
29.Afshar-Oromieh A, Haberkorn U, Eder M, Eisenhut M, Zechmann CM. [68Ga]Gallium-labelled PSMA ligand as superior PET tracer for the diagnosis of prostate cancer: comparison with 18F-FECH. Eur J Nucl Med Mol Imaging. 2012 Jun;39(6):1085-6.
30.von Eyben FE, Picchio M, von Eyben R, Rhee H, Bauman G. (68)Ga-Labeled Prostate-specific Membrane Antigen Ligand Positron Emission Tomography/Computed Tomography for Prostate Cancer: A Systematic Review and Meta-analysis. Eur Urol Focus. 2016 Nov 15.
31.Perera M, Papa N, Christidis D, Wetherell D, Hofman MS, Murphy DG, et al. Sensitivity, Specificity, and Predictors of Positive (68)Ga-Prostate-specific Membrane Antigen Positron Emission Tomography in Advanced Prostate Cancer: A Systematic Review and Meta-analysis. Eur Urol. 2016 Dec;70(6):926-37.
32.Seitz AK, Rauscher I, Haller B, Kronke M, Luther S, Heck MM, et al. Preliminary results on response assessment using (68)Ga-HBED-CC-PSMA PET/CT in patients with metastatic prostate cancer undergoing docetaxel chemotherapy. Eur J Nucl Med Mol Imaging. 2017 Nov 28.
33.Baumann R, Koncz M, Luetzen U, Krause F, Dunst J. Oligometastases in prostate cancer : Metabolic response in follow-up PSMA-PET-CTs after hypofractionated IGRT Oligometastasen bei Prostatakarzinom : Metabolisches Ansprechen in Follow-up-PSMA-PET/CT nach hypofraktionierter bildgefuhrter Strahlentherapie. Strahlenther Onkol. 2017 Nov 27.
34.Pyka T, Okamoto S, Dahlbender M, Tauber R, Retz M, Heck M, et al. Comparison of bone scintigraphy and (68)Ga-PSMA PET for skeletal staging in prostate cancer. Eur J Nucl Med Mol Imaging. 2016 Nov;43(12):2114-21.
35.Rahbar K, Afshar-Oromieh A, Bogemann M, Wagner S, Schafers M, Stegger L, et al. (18)F-PSMA-1007 PET/CT at 60 and 120 minutes in patients with prostate cancer:

biodistribution, tumour detection and activity kinetics. Eur J Nucl Med Mol Imaging. 2018 Mar 14.
36.Onstenk W, de Klaver W, de Wit R, Lolkema M, Foekens J, Sleijfer S. The use of circulating tumor cells in guiding treatment decisions for patients with metastatic castration-resistant prostate cancer. Cancer Treat Rev. 2016 May;46:42-50.
37.de Bono JS, Scher HI, Montgomery RB, Parker C, Miller MC, Tissing H, et al. Circulating tumor cells predict survival benefit from treatment in metastatic castration-resistant prostate cancer. Clin Cancer Res. 2008 Oct 1;14(19):6302-9.
38.Fossa SD, Hess SL, Paus E, Borgen E. Circulating tumor cells in patients with metastatic castration resistant prostate cancer: exploratory findings at a tertiary referral hospital. Res Rep Urol. 2014;6:121-6.
39.Bettegowda C, Sausen M, Leary RJ, Kinde I, Wang Y, Agrawal N, et al. Detection of circulating tumor DNA in early- and late-stage human malignancies. Sci Transl Med. 2014 Feb 19;6(224):224ra24.
40.Diehl F, Li M, Dressman D, He Y, Shen D, Szabo S, et al. Detection and quantification of mutations in the plasma of patients with colorectal tumors. Proc Natl Acad Sci U S A. 2005 Nov 8;102(45):16368-73.
41.Heitzer E, Auer M, Hoffmann EM, Pichler M, Gasch C, Ulz P, et al. Establishment of tumor-specific copy number alterations from plasma DNA of patients with cancer. Int J Cancer. 2013 Jul 15;133(2):346-56.
42.Leary RJ, Kinde I, Diehl F, Schmidt K, Clouser C, Duncan C, et al. Development of personalized tumor biomarkers using massively parallel sequencing. Sci Transl Med. 2010 Feb 24;2(20):20ra14.
43.Dawson SJ, Tsui DW, Murtaza M, Biggs H, Rueda OM, Chin SF, et al. Analysis of circulating tumor DNA to monitor metastatic breast cancer. N Engl J Med. 2013 Mar 28;368(13):1199-209.
44.Hanahan D, Weinberg RA. Hallmarks of Cancer: The Next Generation. Cell. 2011;144:646-74.
45.Pardoll DM. The blockade of immune checkpoints in cancer immunotherapy. Nature Reviews Cancer. 2012;12:252-64.
46.Rijnders M, de Wit R, Boormans JL, Lolkema MPJ, van der Veldt AAM. Systematic Review of Immune Checkpoint Inhibition in Urological Cancers. Eur Urol. 2017 Sep;72(3):411-23.
47.Kantoff PW, Higano CS, Shore ND, Berger ER, Small EJ, Penson DF, et al. Sipuleucel-T Immunotherapy for Castration-Resistant Prostate Cancer. NEJM. 2010;363(5):411-22.
48.Kwon ED, Drake CG, Scher HI, Fizazi K, Bossi A, Van den Eertwegh AJM, et al. Ipilimumab versus placebo after radiotherapy in patients with metastatic castration-resistant prostate cancer that had progressed after docetaxel chemotherapy (CA184-043): a multicentre, randomised, double-blind, phase 3 trial. Lancet Oncol. 2014;15:700-12.
49.Beer TM, Kwon ED, Drake CG, Fizazi K, Logothetis C, Gravis G, et al. Randomized, Double-Blind, Phase III Trial of Ipilimumab Versus Placebo in Asymptomatic or Minimally Symptomatic Patients With Metastatic Chemotherapy-Naive Castration-Resistant Prostate Cancer. J Clin Oncol. 2017 Jan;35(1):40-7.
50.Graff JN, Alumkal JJ, Drake CG, Thomas GV, Redmond WL, Farhad M, et al. Early evidence of anti-PD-1 activity in enzalutamide-resistant prostate cancer. Oncotarget. 2016;7(33):52810-17.
51.Ngwa W, Irabor OC, Schoenfeld JD, Hesser J, Demaria S, Formenti SC. Using immunotherapy to boost the abscopal effect. Nat Rev Cancer. 2018 Feb 16.
52.Formenti SC, Demaria S. Combining radiotherapy and cancer immunotherapy: a paradigm shift. J Natl Cancer Inst. 2013 Feb 20;105(4):256-65.
53.Grass GD, Krishna N, Kim S. The immune mechanisms of abscopal effect in radiation therapy. Curr Probl Cancer. 2016 Jan-Feb;40(1):10-24.
54.Barker HE, Paget JT, Khan AA, Harrington KJ. The tumour microenvironment after radiotherapy: mechanisms of resistance and recurrence. Nat Rev Cancer. 2015 Jul;15(7):409-25.
55.Kepp O, Senovilla L, Vitale I, Vacchelli E, Adjemian S, Agostinis P, et al. Consensus guidelines for the detection of immunogenic cell death. Oncoimmunology. 2014 Oct;3(9):e955691.

56.Lugade AA, Moran JP, Gerber SA, Rose RC, J.G. F, Lord EM. Local radiation therapy of B16 melanoma tumors increases the generation of tumor antigen-specific effector cells that traffic to the tumor. The Journal of Immunology. 2005;174:7516-23.
57.Lugade AA, Sorensen EW, Gerber SA, Moran JP, Frelinger JG, Lord EM. Radiation-Induced IFN-γ Production within the Tumor Microenvironment Influences Antitumor Immunity. Journal of Immunology. 2008;180:3132-39.
58.Golden EB, Pellicciotta I, Demaria S, Barcellos-Hoff MH, Formenti SC. The convergence of radiation and immunogenic cell death signaling pathways. Front Oncol. 2012;2:88.
59.Robinson D, Van Allen EM, Wu YM, Schultz N, Lonigro RJ, Mosquera JM, et al. Integrative clinical genomics of advanced prostate cancer. Cell. 2015 May 21;161(5):1215-28.
60.Goodman AM, Kato S, Bazhenova L, Patel SP, Frampton GM, Miller V, et al. Tumor Mutational Burden as an Independent Predictor of Response to Immunotherapy in Diverse Cancers. Mol Cancer Ther. 2017 Nov;16(11):2598-608.
61.Gibney GT, Weiner LM, Atkins MB. Predictive biomarkers for checkpoint inhibitor-based immunotherapy. Lancet Oncol. 2016;16:e542-51.
62.Malamas AS, Gameiro SR, Knudson KM, Hodge JW. Sublethal exposure to alpha radiation (223Ra dichloride) enhances various carcinomas' sensitivity to lysis by antigen-specific cytotoxic T lymphocytes through calreticulin-mediated immunogenic modulation. Oncotarget. 2016.
63.Kim JW, Shin MS, Kang Y, Kang I, Petrylak DP. Immune Analysis of Radium-223 in Patients With Metastatic Prostate Cancer. Clin Genitourin Cancer. 2017 Oct 24.
64.Bensch F, van der Veen EL, Lub-de Hooge MN, Jorritsma-Smit A, Boellaard R, Kok IC, et al. (89)Zr-atezolizumab imaging as a non-invasive approach to assess clinical response to PD-L1 blockade in cancer. Nat Med. 2018 Dec;24(12):1852-8.
65.Local Guideline EMC 68Ga-PSMA preparation: 'Far: VTGM van Ga-68-PSMA-HBED-CC (Versie 1)'.
66.Veen ELvd, Jorritsma A, Rutgers V, Zijlma R, Luurtsema G, Boersma HH. Investigational medicinal product dossier [89Zr]-Atezolizumab.
