# Supplementary material for: Radium-223 decreases PD-L1 expression in bone metastases: A study with [89Zr]Zr-atezolizumab PET and immunohistological correlation
Source: Eur J Nucl Med Mol Imaging. 2026 Sep 12;53(13):8558–67. doi: 10.1007/s00259-026-08175-9 (PMC13727859; doi:10.1007/s00259-026-08175-9)
Supplement: Supplementary file 3 [file 259_2026_8175_MOESM3_ESM.docx]

**Supplementary Data**

| **Table of Contents** | **Page** |
| --- | --- |
| **Section 1: Supplementary Methods**  Section 1.1: Patients  Section 1.2: Image acquisition and analyses of ^89^Zr-atezolizumab PET/CT  Section 1.3: Flow cytometry of immune cell populations  Section 1.4: Immunohistochemistry of tumor biopsies | 2-4  2  2  2-3  3-4 |
| **Section 2: Supplementary Results**  Section 2.1: Immunohistochemistry shows varying PD-L1 expression in bone metastases  Section 2.2: Abundance of TIM-3+ CD8+ T cells in bone metastases of good responders to radium-223  Section 2.3: Number of circulating lymphocytes declines after radium-223  Section 2.4: Increase in circulating TIM3+ T cells during treatment in good responders to radium-223 | 5-6  5  5  5  5-6 |
| **Section 3: Supplementary Figures**  Figure S1: Study procedures of the Radium223Insight study  Figure S2: Measurement of ^89^Zr-atezolizumab uptake in ^68^Ga-PSMA positive bone lesions  Figure S3: Biodistribution of ^89^Zr-atezolizumab  Figure S4: SUV_max_ of ^89^Zr-atezolizumab  Figure S5: Correlation between ^89^Zr-atezolizumab uptake on PET and PD-L1 expression in tumor biopsies  Figure S6 Example of immunohistochemistry of bone metastasis in a patient with mCRPC  Figure S7: Multiplex immunohistochemistry lymphocyte panel  Figure S8: Quantitative multiplex immunohistochemistry of exhaustion markers expressing CD8+ T cells in bone metastases of patients with mCRPC, treated with radium-223  Figure S9: Quantitative multiplex immunohistochemistry of lymphocytes in bone metastases of patients with mCRPC, treated with radium-223.  Figure S10: Example of immunohistochemistry of bone biopsies  Figure S11: T cell subset frequencies in blood during treatment with radium-223 in patients with mCRPC  Figure S12: Multiplex immunohistochemistry exhaustion panel  Figure S13: Enumeration of immune cell populations in blood  Figure S14: Changes in immune cell populations in blood during treatment with radium-223  Figure S15: T cells with co-inhibitory, co-stimulatory, and chemoattractant receptors  Figure S16: Changes in T cells with frequencies of co-inhibitory, co-stimulatory, and chemoattractant receptors Figure S17: Enumeration of immune cell populations in blood | 7-39  7  8  9  10  11  12  13  14  15  16  17  18  19  20  21-29  30-38  39 |
| **Section 4: Supplementary Tables**  Table S1: Flow cytometry  Table S2: Immunohistochemistry  Table S3: Baseline characteristics  Table S4: Clinical outcomes of evaluable patients  Table S5: Biodistribution of ^89^Zr-atezolizumab | 40-44  40  41  42  43  44 |
| **Section 5: Supplementary References** | 45 |

**Section 1: Supplementary methods**

**1.1. Patients**

Patients were eligible if they met the following criteria: age ≥ 18 years; histologically confirmed prostate cancer; with documented disease progression during androgen deprivation therapy (ADT); baseline PSA level of ≥ 2 ng/ml with evidence of progressively increasing PSA values; prior treatment with at least two other approved agents for metastatic prostate cancer, unless the patient was not able and/or willing to receive such treatment; Eastern Cooperative Oncology Group (ECOG) performance-status score of 0 or 1; estimated life expectancy of six months or longer; and adequate hematologic, renal, and liver function, including platelets >100x10^9^/L, granulocytes >1.5x10^9^/L, and Hb >6.2 mmol/L. Patients were excluded if they had one of the following criteria: prior chemotherapy, other than docetaxel; previous hemibody external radiotherapy or treatment with radioisotopes within the previous 24 weeks; blood transfusion or use of erythropoietin-stimulating agents within the previous four weeks; pathological lymphadenopathy > 1.5 cm in the short-axis diameter on CT or MRI (^68^Ga-PSMA positive lymph nodes < 1.5 cm at baseline were allowed); visceral metastases on CT or MRI; imminent or established spinal cord compression on CT or MRI; or a second active malignancy.

**1.2** **Image acquisition and analyses of ^89^Zr-atezolizumab PET/CT**

^89^Zr-atezolizumab was produced at the University Medical Center Groningen according to the relevant Good Manufacturing Practice guidelines, as described previously (1), and administered intravenously with a predefined dose of 37 MBq (approximately 11 mg atezolizumab, <1% of the therapeutic dose). Imaging was performed seven days after injection to acquire the most optimal target-to-background ratio (1). Patients were imaged from head to mid-thigh on a Biograph mCT scanner (Siemens Healthineers). PET data were acquired in seven bed positions (bp) with 15 min/bp. First, a low-dose non-contrast CT was acquired for attenuation correction and anatomic localization. Following CT, PET images were acquired. PET images were reconstructed using ordered subset expectation maximization (OSEM) with point spread function correction and time-of-flight information using 3 iterations and 21 subsets on a 200x200 matrix. A 3mm Gaussian filter was applied to attenuation corrected images (AC) and 6mm for non-AC images.

For the biodistribution of ^89^Zr-atezolizumab, a 3D sphere with a preset size per organ (brain 4 cm, lung 5 cm, aorta blood pool 2 cm, muscle 5 cm, spleen 4 cm, liver 5 cm, kidney 2 cm, bone marrow 2 cm, intestine 2 cm, bone cortex 1 cm and subcutis 2 cm) was placed in a region of interest without ^68^Ga-PSMA uptake that was suspicious for prostate cancer.

For the measurement of ^89^Zr-atezolizumab uptake in bone metastases, a PERCIST-based lesion selection tool (Hermes Hybrid3D 3.0.1) was used. A tumor mask of ^68^Ga-PSMA positive bone lesions was created for ^68^Ga-PSMA PET/CT images at baseline and after three cycles of radium-223. Detailed analyses of ^68^Ga-PSMA uptake in this patient cohort were described previously (2). Thereafter, an in-house developed image registration tool was used to merge the images of ^89^Zr-atezolizumab PET/CT and ^68^Ga-PSMA PET/CT (**Figure S2**) (2). Within ^68^Ga-PSMA positive bone metastases, ^89^Zr-atezolizumab uptake (SUV_mean_ and SUV_max_) was measured by applying the corresponding predefined tumor mask from the ^68^Ga-PSMA PET/CT to the registered ^89^Zr-atezolizumab PET/CT. Mean ^89^Zr-atezolizumab uptake at different time points was calculated for each patient and lesion. Changes in ^89^Zr-atezolizumab uptake over time were analyzed per patient (total tumor volume) by paired comparisons of the baseline tumor mask and the follow-up tumor mask of the ^89^Zr-atezolizumab PET images. Since bone metastases of mCRPC merge or split frequently over time, changes in ^89^Zr-atezolizumab uptake per lesion could not be reported. To compare ^89^Zr-atezolizumab uptake on PET with PD-L1-expression in tumor biopsies according to IHC, ^89^Zr-atezolizumab PET/CT was merged with the interventional CT of the biopsy procedure. Subsequently, ^89^Zr-atezolizumab uptake (SUV_mean_ of 3D cube of 2 cm) was measured at the biopsy site using the ACCURATE tool (1, 3).

**1.3 Flow cytometry of immune cell populations**

For flow cytometry, blood samples (2x10 ml collected in EDTA tubes) were stored at room temperature, and processed within 24 hours (**Figure S1**). All blood-based immune cell analyses were performed at the Laboratory of Tumor Immunology at EMC. Eighteen immune cell populations were quantified by multicolor flow cytometry of whole blood, as previously described (4, 5). In summary, absolute counts (number of cells/μl blood) of granulocytes (including subsets of eosinophils, mature and immature neutrophils), monocytes (including subsets of dendritic cells (DC), mononuclear myeloid-derived suppressor cells (M-MDSC), classical, intermediate and non-classical monocytes), and lymphocytes (including subsets of B cells, natural killer (NK) cells, T-cells, αβ T-cells and γδ T-cells) were determined. Whole blood was stained using the antibodies specified in **Table S1**, and, after erythrocyte lysis, analyzed by multicolor flow cytometry on a BD 3-laser Celesta flow cytometer with FACSDIVA 8.x software. Absolute cell counts, including lymphocyte, granulocyte and monocyte populations were determined using Flow-Count Fluorospheres (Beckman Coulter).

In addition, frequencies of expression of co-inhibitory receptors (CD-39, LAG3, BTLA, PD-1 and TIM3), co-stimulatory receptors (including CD28, OX40, 4-1BB, CD40L and ICOS), and chemoattractant receptors (CXCR3, CXCR4, CCR1, CCR4 and CCR5) were assessed in subsets of CD4^+^ and CD8^+^ T-cells. To this end, PBMCs were isolated from freshly obtained blood using Ficoll gradient centrifugation and stored according to standardized protocols. Cryopreserved PBMCs were thawed and stained with a mix of antibodies, directed against T-cell markers of co-inhibitory, co-stimulatory and chemoattractant receptors (**Table S1**), as previously described (4, 6). Antibody panels were optimized and compensated using fluorescence minus one (FMO) controls. Measurements were corrected for background fluorescence. Data was gated and analyzed using FlowJo software.

For the enumeration of immune cell populations, freshly obtained blood was used. Eighteen immune cell populations, including (subsets of) granulocytes, monocytes and lymphocytes, were quantified by multicolor flow cytometry, as previously described (**Table S1a**) (4, 5). In addition, frequencies of T cells that express co-inhibitory, co-stimulatory and/or chemoattractant receptors were assessed in subsets of CD4^+^ and CD8^+^ T cells. To this end, cryopreserved peripheral blood mononuclear cells were thawed and stained with a mix of antibodies directed against the T cell markers of interest (**Table S1b**) (4, 6). To assess changes in immune cell populations over time, data from individual patients were normalized, as previously described (5, 7). For clustering and visualization of T-cell subset frequencies, an in-house-written script was used (8).

**1.4 Immunohistochemistry of tumor biopsies**

The biopsy site was chosen based on prior imaging, including ^68^Ga-PSMA PET/CT, clinical judgment, and safety considerations by the interventional radiologist. The obtained tissue was divided into two parts and directly preserved in formalin and liquid nitrogen. Formalin-fixed biopsies were paraffin embedded (FFPE) and decalcified in 10% EDTA solution according to institutional guidelines of the Department of Pathology in EMC. Sections of 4 µm thickness were cut and mounted on glass slides, and assessed for tumor cell presence on HE-stained slides by a pathologist at the RUMC. Subsequently, single PSMA staining (EP192, RUMC), single PD-L1 stainings (SP142, EMC; 22C3, RUMC), single RGB-trichrome staining (EMC), and multiplex IHC (lymphocyte panel (9) and exhaustion panel, RUMC) were performed and analyzed (**Table S2)** (10, 11).

For the PSMA staining, slides were loaded into Dako Omnis (Agilent Dako) for the IHC procedure. Slides were deparaffinized and antigen retrieval was performed by heating to 97 °C in EnVision™ FLEX Target Retrieval Solution, high pH (Agilent Dako, K8004, pH 9) for 30 minutes. Slides were rinsed between steps with EnVision™ FLEX Wash buffer (Agilent Dako, DM831). Peroxidase activity was blocked using EnVision™ FLEX Peroxidase-blocking (Agilent Dako, SM801) for 5 minutes. The slides were incubated with anti-PSMA clone EP192 (AC-0160, Cell Marque, 1:100 dilution) for 60 minutes. Slides were subsequently incubated with EnVision™ FLEX/HRP for 30 minutes. PSMA expression was visualized with EnVision™ FLEX DAB+ chromogen for 10 minutes. Nuclei were counterstained with hematoxylin, and dehydrated with a Prisma Automated Slide Stainer (Sakura Finetek). PD-L1 staining was performed on the automated VENTANA Benchmark ULTRA platform using the SP142 assay and on the Autostainer Link 48 using the 22C3 pharmDx assay, both following manufacturer’s instructions. PSMA and PD-L1 immunoreactivity were evaluated by a pathologist. The PSMA staining was scored based on the degree of expression (score 0-3) and the percentage of positive tumor cells. The PD-L1 staining was separately evaluated for percentage of positive tumor and immune cells (score 0: <1% positive; 1: 1-<5% positive; 2: 5-<50% positive; 3: 50-100% positive). RGB-trichrome staining was applied according to the method described by Gaytan et al. using 1% instead of 0.04% of Fast Green FCF (Sigma-Aldrich/Merck, 861154) and 1% instead of 0.1% Sirius Red (Direct Red 80, Sigma-Aldrich/Merck, 365548) (5).

Multiplex IHC was performed and analyzed as described before, with some changes to the panel (**Table S2**) to be compatible with the PhenoImager HT (Akoya Biosciences) (10). An additional machine learning algorithm was trained using ImmuNet for the detection of PD-1, LAG-3 and TIM-3 on CD8 T cells using ImmuNet (11). PD-L1 staining (E1L3N) within multiplex IHC was visually scored as being negative or positive in three different intensities (1-3) within QuPath.

**Section 2: Supplementary Results**

**2.1. Immunohistochemistry shows varying PD-L1 expression in bone metastases**

After three cycles of radium-223, and at end of treatment, one (33%) and two (100%) biopsies showed PD-L1-expression, respectively. In one patient, PD-L1-expression was negative on tumor cells at all time points, whereas in this patient non-tumor cells only showed low PD-L1-expression at end of treatment. In another patient, high PD-L1-expression on tumor cells at baseline completely disappeared after three cycles of radium-223.

IHC stainings with SP142 and 22C3 (*n*=19) did not show positivity for PD-L1, which may be due to the low PD-L1-expression in mCRPC and technical issues of IHC on decalcified bone metastases. Nevertheless, staining for PD-L1 within the multiplex IHC panel (E1L3N, **Table S2b**) showed PD-L1-expression in 11 (58%, *n*=19) biopsies at baseline.
At re-inspection of the sample with the highest PD-L1-expression (E1L3N), faint positivity was also observed for PD-L1 (22C3). The total PD-L1 score from IHC (E1L3N) showed a positive correlation with ^89^Zr-atezolizumab uptake at the biopsy site (Spearman *r* = 0.348, *n* = 12, *p* = 0.268) **(Figure S5)**.

**2.2. Abundance of TIM-3+ CD8+ T cells in bone metastases of good responders to radium-223**

Median size of the assessed tissue was 7.3 mm^2^ (IQR: 3.1-10.1), including 2.6 mm^2^ (IQR: 0.8-3.8) of tumor tissue and 3.6 mm^2^ (IQR: 1.4-7.0) of bone marrow.

The median number of lymphocytes was lower in tumor tissue than in the surrounding bone marrow (113/mm^2^, IQR: 59-182 *vs.* 239/mm^2^, IQR: 112-356, *p* = 0.003) (**Figure S9a**). Upon quantification of lymphocyte subsets, the median number of cytotoxic T cells and regulatory T cells was higher in tumor than in bone marrow (26/mm^2^ *vs.* 18/mm^2^, *p* = 0.053, and 3/mm^2^ *vs.* 1/mm^2^, *p* = 0.003, respectively), while the number of helper T cells and B cells was lower in tumor than in bone marrow (70/mm^2^ *vs.* 173/mm^2^, *p* = 0.001, and 4/mm^2^ *vs*. 23/mm^2^, *p* < 0.001, respectively) (**Figure S9a**). As a result, the median fraction of cytotoxic T cells and regulatory T cells of the total lymphocyte population was higher in tumor than in bone marrow (25.5% *vs.* 8.3%, *p* < 0.001, and 2.0% *vs*. 0.1%, *p* < 0.001, respectively), while the median fraction of helper T cells and B cells was lower in tumor than in bone marrow (59.6% *vs.* 75.5%, *p* = 0.030, and 3.1 *vs.* 6.0%, *p* = 0.022, respectively) (**Figure S9b**). In tumor and bone marrow, the number of lymphocytes, including all subsets, did not differ between good and poor responders (*p* = 0.481, and *p* = 0.673, respectively). In addition, biopsies were stained for the immune checkpoint receptors PD-1, TIM-3 and LAG-3 and the percentage of single receptor positive CD8^+^ T cells was calculated in tumor and bone marrow (**Figure S6d, S10 and S12**). TIM-3^+^, CD8^+^ T cells and LAG-3^+^, CD8^+^ T cells were more frequently present in bone marrow than in tumor tissue (mean ± SD 24% ± 16% vs. 13% ± 10%, *n* = 17, *p* = 0.010 and 9% ± 10% vs. 2% ± 2%, *n* = 17, *p* = 0.008, respectively, paired T-test), while the frequency of PD-1^+^, CD8^+^ T cells was comparable between the two compartments (mean ± SD 78% ± 13% vs. 78% ± 17%, *n* = 17, *p* = 0.949) (**Figure S10a**). No differences between good and poor responders were seen. However, TIM-3^+^, CD8^+^ T cells tended to be higher in bone marrow of good responders than of poor responders (median (IQR) 25% (10-49%), n = 9 and 16% (11-20%), n = 8, respectively *p* = 0.093, Mann Whitney U Test) (**Figure S10b**).

**2.3: Number of circulating lymphocytes declines after radium-223**

An overview of the absolute quantification of immune cell populations is shown in **Figure S13 and S14**, while all T cell subset frequencies are presented in **Figure S15 and S16**. In summary, the number of eosinophils was higher in good than in poor responders at baseline (median 241 cells/μl blood, IQR: 205-388, *n* = 12 *vs*. 117 cells/μl blood, IQR: 95-167, *n* = 12, *p* = 0.012) (**Figure S17a**). In the total patient cohort, distinct decreases upon treatment with radium-223 were mainly observed in lymphocytes (*p* < 0.001), including all subsets, and mature neutrophils (*p* < 0.001) (**Figures 7b-d and S14**). These decreases tended to be stronger in good than in poor responders.

**2.4: Increase in circulating TIM3+ T cells during treatment in good responders to radium-223**

Although an increase in the frequency of PD-1-expressing CD4^+^ T cells was observed during treatment in poor responders (p < 0.05), the fold change was <1.5 and considered small (**Figure S16**). The frequency of T cells expressing the co-inhibitory receptor LAG-3 (CD223) at baseline was higher in poor than in good responders (CD4^+^: *p* = 0.005 and CD8^+^: *p* = 0.036) (**Figure S14c, d**), while T cells expressing the chemoattractant receptor CXCR3 at baseline were enriched in good responders (CD4^+^: *p* = 0.068 and CD8^+^: *p* = 0.047) (**Figure S14e, f**). Notably, when focusing on on-treatment effects, the frequency of CD4^+^ and CD8^+^ T cells expressing the co-inhibitory receptor TIM-3 (CD366) increased only in good responders, whereas the expression of the co-stimulatory receptor CD40L (CD154) on CD4^+^ T cells decreased in these patients (**Figure S14g-j**).

**Section 3: Supplementary Figures**

**Figure S1 Study procedures in the Radium223Insight study**

Patients were treated with six cycles of radium-223 every four weeks, according to standard-of-care. Blood was collected at baseline, every 1-2 weeks before the next cycle, at end of treatment, during follow-up and at time of treatment failure. Biopsies and imaging were performed at baseline, after three cycles of radium-223, at end of treatment and at time of treatment failure. In the current manuscript, the results of ^68^Ga-PSMA PET/CT, ^89^Zr-atezolizumab PET/CT, and immune analyses in blood and tissue are described. The results of response evaluation with ^68^Ga-PSMA PET/CT and liquid biopsies were described previously (2, 12).

**Figure S2 Measurement of ^89^Zr-atezolizumab uptake in ^68^Ga-PSMA positive bone lesions
A)** To create a reference standard for tumor lesions, ^68^Ga-PSMA positive bone lesions were semi-automatically selected, resulting in a tumor mask (green stripes) for each ^68^Ga-PSMA PET/CT. **B)** To correct for differences in patient position between the two PET/CTs (illustrated in pink and green), ^89^Zr-atezolizumab PET/CT was registered to ^68^Ga-PSMA PET/CT using an in-house developed image registration tool. This tool enables the alignment of rigid structures, such as bones, of multiple PET/CTs. **C)** The tumor mask created in A) was applied to the registered ^89^Zr-atezolizumab PET/CT in B) to measure uptake of ^89^Zr-atezolizumab in ^68^Ga-PSMA positive lesions (green stripes).

**Figure S3 Biodistribution of ^89^Zr-atezolizumab during treatment with radium-223 in patients with mCRPC
A)** ^89^Zr-atezolizumab uptake in multiple organs at baseline (*n* = 17). SUV_mean_ was measured in a 3D sphere with a preset size per organ in a region of interest without ^68^Ga-PSMA uptake, suspected of prostate cancer, using the ACCURATE tool. **B)** Comparison of ^89^Zr-atezolizumab uptake in multiple organs between baseline and after three cycles of radium-223 (*n* = 9), using a Wilcoxon signed-rank test. * represents a p-value ≤ 0.05. Boxplots illustrate median, interquartile range, and range.

**Figure S4 SUV_max_ of ^89^Zr-atezolizumab in bone metastases during treatment with radium-223 in patients with mCRPC
A)** SUV_max_ of ^89^Zr-atezolizumab in ^68^Ga-PSMA positive bone lesions. Boxplots illustrate median, interquartile range and range of uptake in all lesions within each individual patient at baseline (*n* = 17) and after three cycles of radium-223 (*n* = 8). **B)** Change in SUV_max_ of ^89^Zr-atezolizumab from baseline to after three cycles of radium-223 for patients with complete imaging at both time points (*n* = 8), Wilcoxon signed-rank test. **C)** Change in SUV_max_ of ^68^Ga-PSMA from baseline to after three cycles of radium-223 for patients with complete imaging at both time points (*n* = 8), Wilcoxon signed-rank test. **D)** Comparison of SUV_max_ of ^89^Zr-atezolizumab between good and poor responders to radium-223 (*n* = 7 and 8, respectively) at baseline, using a Mann-Whitney U test. **E)** Comparison of change in SUV_max_ of ^89^Zr-atezolizumab from baseline to after three cycles of radium-223 between good and poor responders (*n* = 4 and 3, respectively), using a Mann-Whitney U test.

**Figure S5 Correlation between ^89^Zr-atezolizumab uptake on PET and PD-L1 expression in tumor biopsies**
^89^Zr-atezolizumab uptake on PET at the biopsy site and PD-L1 expression from immunohistochemistry of tumor biopsies were correlated using a Spearman correlation test.

**Figure S6 Example of immunohistochemistry of bone metastasis in a patient with mCRPC. A-C)**
Slides (magnification 40x) of a biopsy of bone metastasis at baseline of one of the included patients, stained with **A)** hematoxylin-eosin (HE), **B)** prostate specific membrane antigen (PSMA), positive in 100% of the tumor cells, **C)** RGB-trichrome (mineralized bone in green, osteoid in red, cartilage in blue), **D/E)** show images of multiplex immunohistochemistry for T cell exhaustion markers and subsets of lymphocytes and tumor cells of a biopsy of bone metastasis at baseline.

**Figure S7 Multiplex immunohistochemistry lymphocyte panel**

**A)** Multiplex stained slides for the lymphocyte panel were scanned at 20× magnification and images for analysis were selected using Phenochart. **B)** Images were unmixed using inForm to visualize the markers: CD3 (red), FOXP3 (green), CD8 (cyan), CD20 (magenta), CD56 (yellow), tumor (white), DAPI (blue) and autofluorescence (not shown). **C)** A tissues segmentation algorithm was trained with inForm to distinguish between areas with tumor, stroma and background. **D)** Lymphocytes were identified based on marker expression using a machine-learning algorithm called ImmuNet. Colors show inferred intensity of surface marker expression for cells detected by the algorithm. **E)** Lymphocytes that are recognized by the machine-learning algorithm were exported as FCS file and were phenotyped based on inferred marker expression per tumor and stroma tissue region. Using a gating strategy that first separated T-cells (CD3^+^CD20^-^) from B-cells (CD3^-^CD20^+^). Next, T cells were further separated into cytotoxic T cells (CD3^+^CD8^+^FOXP3^-^), regulatory T cells (CD3^+^CD8^-^FOXP3^+^) and helper T cells (CD3^+^CD8^-^FOXP3^-^). From the CD3^-^CD20^-^ events NK cells were gated out based on the CD56 signal. FCS = flow cytometry standard.

**Figure S8 Quantitative multiplex immunohistochemistry of exhaustion markers expressing CD8^+^ T cells in bone metastases of patients with mCRPC, treated with radium-223.**

Slides were divided in tumor tissue and bone marrow by automatic tumor segmentation based on a set of tumor marker stainings (pan-cytokeratin, PSMA and EpCAM). **A)** Comparison of the fraction of CD8^+^ T cells, that express either PD-1, TIM-3 or LAG-3, in tumor tissue or bone marrow (n = 17, two biopsies were excluded due to absence of CD8^+^ T cells in the tumor tissue), using a paired T-test. **B)** Comparison of the fraction of CD8^+^ T cells, that either express PD-1, TIM-3 or LAG-3, in tumor tissue or bone marrow between good and poor responders to radium-223 (*n* = 9 and *n* = 8, respectively), using a Mann-Whitney U test. **C)** Fraction of CD8^+^ T cells, that express either PD-1, TIM-3 or LAG-3, in tumor tissue or bone marrow for two patients, who had paired biopsies at baseline and during treatment. Boxplots illustrate median, interquartile range and range. BL = baseline, 12w = after three cycles of radium-223, EoT = end of treatment, BM = bone marrow. One, two and three stars represent p-values ≤ 0.05, ≤ 0.01 and ≤ 0.001, respectively.

**Figure S9 Quantitative multiplex immunohistochemistry of lymphocytes in bone metastases of patients with mCRPC, treated with radium-223.**

Slides were divided into tumor tissue and bone marrow by automatic tumor segmentation based on a set of tumor marker stainings (pan-cytokeratin, PSMA and EpCAM). **A)** Comparison of the number of cells/mm^2^ in tumor tissue and bone marrow at baseline (*n* = 19) for multiple lymphocyte subsets, using a Wilcoxon signed-rank test. **B)** Fractions of lymphocyte subsets of the total lymphocyte population in tumor tissue and bone marrow at baseline (*n* = 19), compared with a Wilcoxon signed-rank test. **C)** Number of cells/mm^2^ of lymphocyte subsets in tumor tissue and bone marrow for two patients, who had paired biopsies at baseline and during treatment. Boxplots illustrate median, interquartile range and range. BL = baseline, 12w = after three cycles of radium-223, EoT = end of treatment, BM = bone marrow. One, two and three stars represent p-values ≤ 0.05, ≤ 0.01 and ≤ 0.001, respectively.

**Figure S10 Example of immunohistochemistry of bone biopsies in a patient with mCRPC during radium-223**
Slides stained with hematoxylin-eosin (HE), prostate-specific membrane antigen (PSMA) and RGB-trichrome (mineralized bone in green, osteoid in red, cartilage in blue) are 100x magnified. The bottom row shows images of multiplex immunohistochemistry for subsets of lymphocytes and tumor cells. The colors represent stainings for DAPI (blue), CD20 (pink), CD3 (red), FOXP3 (green), CD56 (yellow), CD8 (blue), and tumor markers (white). The scale bar represents 100 μm.

**Figure S11 T cell subset frequencies in blood during treatment with radium-223 in patients with mCRPC.**

Comparison of frequencies of CD4^+^ and CD8^+^ T cells expressing either PD-1 (CD279) **(A/B)**, LAG-3 (CD223) **(C/D)** or CXCR3 **(E/F)** at baseline between good and poor responders to radium-223, using a Mann-Whitney U test. **G-J)** Change in frequencies in CD4^+^ and CD8^+^ T cells expressing eitherTIM-3 (CD366)**(G/H)** or CD40L (CD154)**(I/J)** during treatment in good and poor responders to radium-223, using a Wilcoxon signed-rank test. For good (green) and poor responders (red), *n* = 13, 13, 13 and 11, and *n* = 12, 10, 8 and 0, at baseline, before the second and fourth cycle and at end of treatment, respectively. Boxplots illustrate median, interquartile range and range. G = good responder, P = poor responder, EoT = end of treatment.

**Figure S12 Multiplex immunohistochemistry exhaustion panel**

**A)** Multiplex stained slides for the exhaustion panel were scanned at 20× magnification and images for analysis were selected using Phenochart. Images were unmixed using inForm to visualize the markers: CD8 (red), PD-L1 (green), PD-1 (cyan), LAG-3 (magenta), TIM-3 (yellow), tumor (white), DAPI (blue) and autofluorescence (not shown). **B)** Cells expressing markers were identified based on marker expression using a machine-learning algorithm called ImmuNet. Colors show inferred intensity of surface marker expression for cells detected by the algorithm. **C)** Cells that are recognized by the machine-learning algorithm were exported as FCS file and were phenotyped based on inferred marker expression per tumor and stroma tissue region. Using a gating strategy, CD8^+^ T-cells were selected first. Next, thresholds for positive expression of PD-1,TIM-3 and LAG-3 were applied to the CD8^+^ T cells per tumor and stroma tissue region.

**Figure S13 Enumeration of immune cell populations in blood during treatment with radium-223 in patients with mCRPC**
Comparison of (subsets of) granulocytes, monocytes and lymphocytes in blood between good and poor responders at baseline and during treatment with radium-223, using a Mann-Whitney U test (*n* = 12, 11, 12 and 11, and *n* = 12, 8, 8 and 0, at baseline, before the second and fourth cycle and at end of treatment, respectively). *, ** and *** represent p-values ≤ 0.05, ≤ 0.01 and ≤ 0.001, respectively. Boxplots illustrate median, interquartile range, and range. EoT = end of treatment.

**Figure S14 Changes in immune cell populations in blood during treatment with radium-223 in patients with mCRPC**
Changes in normalized immune cell population counts, including subsets of granulocytes, monocytes and lymphocytes, during treatment with radium-223 for good and poor responders (*n* = 12, 11, 12 and 11, and *n* = 12, 8, 8 and 0, at baseline, before the second and fourth cycle and at end of treatment, respectively). Wilcoxon signed-rank tests were performed between baseline and before cycle four for good and poor responders separately. One, two, and three stars represent p-values ≤ 0.05, ≤ 0.01 and ≤ 0.001, respectively. Boxplots illustrate median, interquartile range and range. EoT = end of treatment.

**Figure S15 T cells with co-inhibitory, co-stimulatory and chemoattractant receptors during treatment with radium-223 in patients with mCRPC**	Comparison of single (**A, D and G**) and double positive (**B, C, E, F, H and I**) T cell subset frequencies in CD4^+^ and CD8^+^ T cells between good and poor responders to radium-223, using a Mann-Whitney U test. For good and poor responders, *n* = 13, 13, 13 and 11, and *n* = 12, 10, 8 and 0, at baseline, before the second and fourth cycle, and at end of treatment, respectively. Boxplots illustrate median, interquartile range and range. G = good responder, P = poor responder, EoT = end of treatment.

**A) Comparison of single T cell subset frequencies of co-inhibitory receptors in CD4^+^ and CD8^+^ T cells in good and poor responders**

**B) Comparison of double T cell subset frequencies of co-inhibitory receptors in CD4^+^ T cells in good and poor responders**

**C) Comparison of double T cell subset frequencies of co-inhibitory receptors in CD8^+^ T cells in good and poor responders**

**D) Comparison of single T cell subset frequencies of co-stimulatory receptors in CD4^+^ and CD8^+^ T cells in good and poor responders**

**E) Comparison of double T cell subset frequencies of co-stimulatory receptors in CD4^+^ T cells in good and poor responders**

**F) Comparison of double T cell subset frequencies of co-stimulatory receptors in CD8^+^ T cells in good and poor responders**

**G) Comparison of single T cell subset frequencies of chemoattractant receptors in CD4^+^ and CD8^+^ T cells in good and poor responders**

**H) Comparison of double T cell subset frequencies of chemoattractant receptors in CD4^+^ T cells in good and poor responders**

**I) Comparison of double T cell subset frequencies of chemoattractant receptors in CD8^+^ T cells in good and poor responders**

**Figure S16 Changes in T cells with frequencies of co-inhibitory, co-stimulatory and chemoattractant receptors during treatment with radium-223 in patients with mCRPC**.

Changes in single (**A, D and G**) and double positive (**B, C, E, F, H and I**) T cell subset frequencies in CD4^+^ and CD8^+^ T cells during treatment in good and poor responders to radium-223, using a Wilcoxon signed-rank test. For good and poor responders, *n* = 13, 13, 13 and 11, and *n* = 12, 10, 8 and 0, at baseline, before the second and fourth cycle and at end of treatment, respectively. Boxplots illustrate median, interquartile range and range. G = good responder, P = poor responder, EoT = end of treatment.

**A) Changes in single positive co-inhibitory receptor T cell subset frequencies in CD4^+^ and CD8^+^ T cells during treatment in good and poor responders to radium-223.**

**B) Changes in double positive co-inhibitory receptor T cell subset frequencies in CD4^+^ T cells during treatment in good and poor responders to radium-223.**

**C) Changes in double positive co-inhibitory receptor T cell subset frequencies in CD8^+^ T cells during treatment in good and poor responders to radium-223.**

**D) Changes in single positive co-stimulatory receptor T cell subset frequencies in CD4^+^ and CD8^+^ T cells during treatment in good and poor responders to radium-223.**

**E) Changes in double positive co-stimulatory receptor T cell subset frequencies in CD4^+^ T cells during treatment in good and poor responders to radium-223.**

**F) Changes in double positive co-stimulatory receptor T cell subset frequencies in CD8^+^ T cells during treatment in good and poor responders to radium-223.**

**G) Changes in single positive chemoattractant receptor T cell subset frequencies in CD4^+^ and CD8^+^ T cells during treatment in good and poor responders to radium-223.**

**H) Changes in double positive chemoattractant receptor T cell subset frequencies in CD4^+^ T cells during treatment in good and poor responders to radium-223.**

**I) Changes in double positive chemoattractant receptor T cell subset frequencies in CD8^+^ T cells during treatment in good and poor responders to radium-223.**

**Figure S17 Enumeration of immune cell populations in blood.**

**A)** Comparison of absolute eosinophil counts in blood between good (green) and poor (red) responders at baseline and during treatment with radium-223, using a Mann-Whitney U test. **B-D)** Changes in absolute immune cell population counts, including subsets of granulocytes **(B)**, monocytes **(C)** and lymphocytes **(D)**, during treatment with radium-223 for good (green) and poor (red) responders, using a Wilcoxon signed-rank test. Exact phenotypes of described immune cell populations are summarized in Table S1a. *, ** and *** represent p-values ≤ 0.05, ≤ 0.01 and ≤ 0.001, respectively. Boxplots illustrate median, interquartile range, and range. For good and poor responders, *n* = 12, 11, 12 and 11, and *n* = 12, 8, 8 and 0, at baseline, before the second and fourth cycle and at end of treatment, respectively. EoT = end of treatment.

### **Section 4: Supplementary Tables**

**Table S1a Flow cytometry absolute immune cell populations panel**

| **Flow cytometry** | **Subset** | **Marker** | **Subset** | **Marker** |
| --- | --- | --- | --- | --- |
| **Enumeration of absolute immune cell populations** | Granulocytes | CD45^+^ SSC | Eosinophils | CD15^+^ / CD16^-^ |
|  |  |  | Mature neutrophils | CD15^high^ / CD16^high^ |
|  |  |  | Immature neutrophils | CD15^+^ / CD16^+^ |
|  | Monocytes | CD45^+^ SSC | Classical monocytes | CD14^+^ / CD16^-^ |
|  |  |  | Intermediate monocytes | CD14^+^ / CD16^+^ |
|  |  |  | Non-classical monocytes | CD14^-^ / CD16^+^ |
|  |  |  | DCs | CD14^-^ / CD16^-^ / CD11c^-^ |
|  |  |  | M-MDSCs | CD14^+^ / CD16^-^ / CD11b^+^ / HLA-DR^low^ |
|  | Lymphocytes | CD45^+^ SSC | B cells | CD3^-^ / CD19^+^ |
|  |  |  | NK cells | CD3^-^ / CD56^+^ / CD16^+/-^ |
|  |  |  | T cells | CD3^+^ |
|  |  |  | αβ T cells | CD3^+^ / TCRαβ^+^ / CD4^+^ or CD8^+^ |
|  |  |  | γδ T cells | CD3^+^ / TCRγδ^+^ |

### **Table S1b Flow cytometry T cell subset panel**

| **Flow cytometry** | **Subset** | **Marker** |
| --- | --- | --- |
| **T cell subset frequencies** | T cell co-inhibitory receptors | CD57, LAG3 (CD223), BTLA (CD272), PD-1 (CD279), TIM3 (CD366) |
|  | T cell co-stimulatory receptors | CD28, OX40 (CD134), 4-1BB (CD137), CD40L, ICOS (CD278) |
|  | T cell chemoattractant receptors | CXCR3 (CD183), CXCR4 (CD184), CCR1 (CD191), CCR4 (CD194), CCR5 (CD195) |

**Table S2a Multiplex IHC lymphocyte panel**

| **Order** | **Antibody target** | **Clone** | **Catalogue number** | **Manufacturer** | **Dilution** | **Opal dye** |
| --- | --- | --- | --- | --- | --- | --- |
| 1 | CD56 | MRQ-42 | 156R-94 | Cell Marque | 1/1500 | 620 |
| 2 | CD8 | C8/144B | M7103 | Agilent Dako | 1/200 | 690 |
| 3 | CD20 | L26 | MS-340-S | Thermo Scientific | 1/300 | 480 |
| 4 | CD3 | Sp7 | RM-9107 | Thermo Scientific | 1/200 | 520 |
| 5 | FOXP3 | 236A/E7 | 14-4777-37 | Thermo Scientific | 1/100 | 570 |
| 6 | Pan-cytokeratin  PSMA  EpCAM | AE1/AE3+5D3  EP192  VU-1D9 | ab86734  BSB6349  ab187372 | Abcam  Bio SB  Abcam | 1/1000  1/1000  1/1000 | 780 |

**Table S2b Multiplex IHC exhaustion panel**

| **Order** | **Antibody target** | **Clone** | **Catalogue number** | **Manufacturer** | **Dilution** | **Opal dye** |
| --- | --- | --- | --- | --- | --- | --- |
| 1 | PD-1 | D4W2J | 86163 | Cell Signaling | 1/100 | 620 |
| 2 | CD8 | C8/144B | M7103 | Agilent Dako | 1/1600 | 520 |
| 3 | PD-L1 | E1L3N | 13684 | Cell Signaling | 1/500 | 480 |
| 4 | LAG3 | EPR4392 | ab180187 | Abcam | 1/5000 | 520 |
| 5 | TIM3 | 2321C | MAB23652 | R&D systems | 1/50 | 570 |
| 6 | Pan-cytokeratin  PSMA  EpCAM | AE1/AE3+5D3  EP192  VU-1D9 | ab86734  BSB6349  ab187372 | Abcam  Bio SB  Abcam | 1/1000  1/1000  1/1000 | 780 |

**Table S3 Baseline characteristics of evaluable patients**	SD = standard deviation, IQR = interquartile range, ADT = androgen deprivation therapy, ECOG = Eastern Cooperative Oncology Group, PSA = prostate specific antigen, ALP = alkaline phosphatase, LDH = lactate dehydrogenase, Hb = hemoglobin, WBC = white blood cells, ANC = absolute neutrophil count.

| **Characteristic** | **Patients**  N = 27 |
| --- | --- |
| **Age in years** | |
| *Mean ± SD* | 70.1 ± 8.2 (n=27) |
| **Histopathology at diagnosis** |  |
| *Adenocarcinoma* | 21 (78%) |
| *Adenocarcinoma with focal neuroendocrine differentiation* | 1 (4%) |
| *Not available* | 5 (19%) |
| **Number of prior treatment lines** | |
| *Median (IQR)* | 2 (1-2) (n=27) |
| **Prior systemic therapies for metastatic prostate cancer** | |
| *Abiraterone* | 13 (48%) |
| *Enzalutamide* | 15 (56%) |
| *Docetaxel* | 16 (59%) |
| *Other treatment* | 4 (15%) |
| **ECOG performance status score** | |
| *0* | 12 (44%) |
| *1* | 15 (56%) |
| **Laboratory values (median (IQR))** | |
| PSA (ug/L) | 50 (15-120) (n=27) |
| ALP (U/L) | 148 (115-272) (n=27) |
| *LDH (U/L)* | 230 (194-262) (n=27) |
| *Hb (mmol/L)* | 7.8 (7.1-8.4) (n=27) |
| *WBC (10^9^/L)* | 7.2 (5.1-7.9) (n=26) |
| *ANC (10^9^/L)* | 4.8 (3.1-5.5) (n=21) |
| *Platelets (10^9^/L)* | 239 (207-282) (n=27) |
| **Extent of bone metastases** | |
| *<6* | 1 (4%) |
| *6-20* | 13 (48%) |
| *>20* | 12 (44%) |
| *Undetermined due to missing bone scintigraphy* | 1 (4%) |

**Table S4 Clinical outcomes of evaluable patients**
IQR = interquartile range. PSA = prostate specific antigen. ALP = alkaline phosphatase.

| **Clinical outcome** | **Patients**  N = 27 |
| --- | --- |
| **Number of received radium-223 injections** | |
| *1-3* | 7 (26%) |
| *4-5* | 7 (26%) |
| *6* | 13 (48%) |
| **Failure free survival (weeks)** | |
| *Median (IQR)* | 22.9 (15.0-25.9) |
| **Reason for treatment failure** |  |
| *Progression of bone disease* | 12 (44%) |
| *Development of extra-osseous metastases (+/- progression of bone disease)* | 12 (44%) |
| *Adverse events* | 2 (7%) |
| *Progression of disease, not specified* | 1 (4%) |
| **Overall survival (weeks)** |  |
| *Median (IQR)* | 48.0 (27.0-91.0) |
| **ALP response after three cycles of radium-223 (≤ -30% from baseline)** | |
| *Yes* | 13 (48%) |
| *No* | 12 (44%) |
| *Unspecified, due to treatment discontinuation before three cycles* | 2 (7%) |
| **PSA response after three cycles of radium-223 (≤ -30% from baseline)** | |
| *Yes* | 2 (7%) |
| *No* | 23 (85%) |
| *Unspecified, due to treatment discontinuation before three cycles* | 2 (7%) |
| **Time to ALP progression (weeks)** | |
| *Median (IQR)* | 39.9 (28.7-NR) |
| **Time to PSA progression (weeks)** | |
| *Median (IQR)* | 15.0 (14.7-15.9) |

**Table S5 Biodistribution of ^89^Zr-atezolizumab in patients with mCRPC**
SUV_mean_ of ^89^Zr-atezolizumab in the spleen, liver, kidney, aorta, intestine, lung, bone cortex, bone marrow, muscle, subcutis and brain. Biodistribution did not change from baseline to after three cycles of radium-223 for all organs, except liver (*p* = 0.038), subcutis (*p* = 0.011) and brain (*p* = 0.021). Clinical reasons for the increased uptake in liver, i.e. metastases or signs of hepatitis, were not observed for the three patients with aberrant values after three cycles of radium-223. Changes in the biodistribution of the subcutis and brain were not considered relevant, as the absolute SUV_mean_ values were extremely low.

| **Organ** | **SUV_mean_ ^89^Zr-atezolizumab (median, IQR)** | | | **P-value** |
| --- | --- | --- | --- | --- |
|  | *Baseline*  *n = 17* | *Baseline for patients with follow-up PET*  *n = 9* | *After three cycles of radium-223*  *n = 9* | *Baseline vs. after three cycles of radium-223 for patients with paired data* |
| Spleen | 12.7 (10.0-16.2) | 11.3 (6.5-16.7) | 11.2 (6.9-12.9) | 0.173 |
| Liver | 4.9 (4.4-6.1) | 4.6 (4.0-5.5) | 6.0 (5.6-12.5) | 0.038 |
| Kidney | 3.0 (2.3-3.8) | 3.0 (2.3-4.1) | 2.5 (1.7-2.9) | 0.173 |
| Aorta | 1.9 (1.4-2.5) | 2.4 (1.9-2.6) | 2.2 (2.0-2.7) | 0.953 |
| Intestine | 1.7 (1.2-2.4) | 1.8 (1.1-2.4) | 1.6 (1.0-2.6) | 0.767 |
| Lung | 0.9 (0.7-1.2) | 0.9 (0.7-1.2) | 0.7 (0.6-1.3) | 0.767 |
| Bone cortex | 0.5 (0.3-0.8) | 0.5 (0.3-0.8) | 0.4 (0.3-0.8) | 0.859 |
| Bone marrow | 0.5 (0.3-0.8) | 0.5 (0.3-0.6) | 0.4 (0.3-0.7) | 0.767 |
| Muscle | 0.3 (0.2-0.4) | 0.4 (0.2-0.4) | 0.3 (0.2-0.4) | 0.678 |
| Subcutis | 0.2 (0.2-0.3) | 0.3 (0.2-0.4) | 0.1 (0.1-0.2) | 0.011 |
| Brain | 0.1 (0.1-0.1) | 0.1 (0.1-0.1) | 0.1 (0.0-0.1) | 0.021 |

**Section 5: Supplementary References**

1. Bensch F, van der Veen EL, Lub-de Hooge MN, Jorritsma-Smit A, Boellaard R, Kok IC, et al. (89)Zr-atezolizumab imaging as a non-invasive approach to assess clinical response to PD-L1 blockade in cancer. Nat Med. 2018;24(12):1852-8.

2. de Jong AC, Segbers M, Ling SW, Graven LH, Mehra N, Hamberg P, et al. (68)Ga-PSMA PET/CT for Response Evaluation of (223)Ra Treatment in Metastatic Prostate Cancer. J Nucl Med. 2023.

3. Boellaard R. Quantitative oncology molecular analysis suite: ACCURATE. J Nucl Med. 2018;59:1753.

4. Kunert A, Basak EA, Hurkmans DP, Balcioglu HE, Klaver Y, van Brakel M, et al. CD45RA(+)CCR7(-) CD8 T cells lacking co-stimulatory receptors demonstrate enhanced frequency in peripheral blood of NSCLC patients responding to nivolumab. J Immunother Cancer. 2019;7(1):149.

5. Rijnders M, Robbrecht DGJ, Oostvogels AAM, van Brakel M, Boormans JL, Aarts MJB, et al. A blood-based immune marker for resistance to pembrolizumab in patients with metastatic urothelial cancer. Cancer Immunol Immunother. 2023;72(3):759-67.

6. Rijnders M, Balcioglu HE, Robbrecht DGJ, Oostvogels AAM, Wijers R, Aarts MJB, et al. Anti-PD-1 Efficacy in Patients with Metastatic Urothelial Cancer Associates with Intratumoral Juxtaposition of T Helper-Type 1 and CD8(+) T cells. Clin Cancer Res. 2022;28(1):215-26.

7. Creemers JHA, van der Doelen MJ, van Wilpe S, Hermsen R, Duiveman-de Boer T, Somford DM, et al. Immunophenotyping Reveals Longitudinal Changes in Circulating Immune Cells During Radium-223 Therapy in Patients With Metastatic Castration-Resistant Prostate Cancer. Front Oncol. 2021;11:667658.

8. Mahajan S, Balcioglu HE, Oostvogels A, Dik WA, Chan KCA, Lo KW, et al. Frequency of peripheral CD8+ T cells expressing chemo-attractant receptors CCR1, 4 and 5 increases in NPC patients with 3EBV clearance upon radiotherapy. Submitted.

9. van Wilpe S, Simnica D, Slootbeek P, van Ee T, Pamidimarri Naga S, Gorris MAJ, et al. Homologous recombination repair deficient prostate cancer represents an immunologically distinct subtype. Oncoimmunology. 2022;11(1):2094133.

10. Gorris MAJ, Martynova E, Sweep MWD, van der Hoorn IAE, Sultan S, Claassens M, et al. Multiplex Immunohistochemical Analysis of the Spatial Immune Cell Landscape of the Tumor Microenvironment. J Vis Exp. 2023(198).

11. Sultan S, Gorris MAJ, Martynova E, van der Woude LL, Buytenhuijs F, van Wilpe S, et al. ImmuNet: a segmentation-free machine learning pipeline for immune landscape phenotyping in tumors by multiplex imaging. Biol Methods Protoc. 2025;10(1):bpae094.

12. de Jong AC, Isebia KT, Ling SW, de Weerd V, Van NM, Kraan J, et al. Liquid Biopsies for Early Response Evaluation of Radium-223 in Metastatic Prostate Cancer. JCO Precis Oncol. 2023;7:e2300156.
